# Agonist-Induced Ca^2+^ Signaling in HEK-293-Derived Cells Expressing a Single IP_3_ Receptor Isoform

**DOI:** 10.3390/cells13070562

**Published:** 2024-03-22

**Authors:** Ekaterina N. Kochkina, Elizaveta E. Kopylova, Olga A. Rogachevskaja, Nina P. Kovalenko, Natalia V. Kabanova, Polina D. Kotova, Marina F. Bystrova, Stanislav S. Kolesnikov

**Affiliations:** Institute of Cell Biophysics, Pushchino Scientific Center for Biological Research of the Russian Academy of Sciences, 3 Institutskaya Street, 142290 Pushchino, Russia

**Keywords:** IP_3_ receptor, Ca^2+^ signaling, CRISP/Cas9, R-CEPIA1er, Ca^2+^ imaging

## Abstract

In mammals, three genes encode IP_3_ receptors (IP_3_Rs), which are involved in agonist-induced Ca^2+^ signaling in cells of apparently all types. Using the CRISPR/Cas9 approach for disruption of two out of three IP_3_R genes in HEK-293 cells, we generated three monoclonal cell lines, IP3R1-HEK, IP3R2-HEK, and IP3R3-HEK, with the single functional isoform, IP_3_R1, IP_3_R2, and IP_3_R3, respectively. All engineered cells responded to ACh with Ca^2+^ transients in an “all-or-nothing” manner, suggesting that each IP_3_R isotype was capable of mediating CICR. The sensitivity of cells to ACh strongly correlated with the affinity of IP_3_ binding to an IP_3_R isoform they expressed. Based on a mathematical model of intracellular Ca^2+^ signals induced by thapsigargin, a SERCA inhibitor, we developed an approach for estimating relative Ca^2+^ permeability of Ca^2+^ store and showed that all three IP_3_R isoforms contributed to Ca^2+^ leakage from ER. The relative Ca^2+^ permeabilities of Ca^2+^ stores in IP3R1-HEK, IP3R2-HEK, and IP3R3-HEK cells were evaluated as 1:1.75:0.45. Using the genetically encoded sensor R-CEPIA1er for monitoring Ca^2+^ signals in ER, engineered cells were ranged by resting levels of stored Ca^2+^ as IP3R3-HEK ≥ IP3R1-HEK > IP3R2-HEK. The developed cell lines could be helpful for further assaying activity, regulation, and pharmacology of individual IP_3_R isoforms.

## 1. Introduction

Extracellular cues regulate diverse cellular functions by involving a variety of surface receptors and intracellular signaling pathways. The mobilization of intracellular Ca^2+^ is central to transduction of many first messengers that act through G-protein-coupled receptors (GPCRs) and receptor tyrosine kinases (RTKs) coupled to the phosphoinositide cascade [1,2,3]. GPCRs stimulate the phosphoinositide cascade by involving the β1–4 isoforms of phospholipase C (PLC) in a αGq- and/or βγGi-dependent manner, while RTKs employ PLCγ [2,3,4,5,6]. Once stimulated, PLC produces two second messengers, IP_3_ and diacylglycerol, by hydrolyzing the precursor lipid phosphatidylinositol 4,5-bisphosphate. The primary mode of action of IP_3_ is to release Ca^2+^ from Ca^2+^ store through IP_3_ receptors (IP_3_Rs) [1,3]. Three genes encode IP_3_R subunits IP_3_R1, IP_3_R2, and IP_3_R3, which form primarily homotetrameric IP_3_-gated Ca^2+^ channels in the endoplasmic reticulum (ER). Moreover, evidence exists that different IP_3_R subunits also can form heterotetrameric complexes with specific functional and regulatory properties [3,7,8,9,10]. This and alternative splicing of the IP_3_R genes increase the functional heterogeneity of IP_3_-gated channels, posing an additional complexity to studying physiological functions and regulations of IP_3_Rs in cells [11,12].

Most cell types express two or even all three IP_3_R genes [12], implying that any particular IP_3_R subtype cannot cover the reported diversity of intracellular IP_3_/Ca^2+^ signaling [1]. The expression pattern of IP_3_Rs is not uniform among different tissues and cell types, suggesting a specific role for individual IP_3_R isoforms or their combinations in cell physiology [12]. For instance, IP_3_R1 predominates in Purkinje neurons, cardiac myocytes rely largely on IP_3_R2, and insulin-secreting β-cells, taste cells, and vomeronasal sensory neurons express primarily IP_3_R3 [13,14,15,16,17].

Since agonist-induced IP_3_/Ca^2+^ signaling is pivotal to the physiology of apparently all cell types, IP_3_Rs were subjected to intensive studies at molecular, biophysical, and functional levels [1,3,9,10,18]. Reportedly, all IP_3_R isoforms are regulated by cytosolic Ca^2+^ in a bimodal manner, suggesting that IP_3_Rs hold two allosteric Ca^2+^-binding sites, both activatory and inhibitory [1,3,19,20]. Although the activatory site has been identified, the structure and location of the inhibitory site is still debatable [3]. Characteristic of IP_3_R regulation is that IP_3_ binding increases affinity of the activatory site, and its occupation by Ca^2+^ increases the open probability of the IP_3_-gated channel [19]. Owing to this interdependent regulation of IP_3_R gating by the primary co-agonists, Ca^2+^ ions released from the ER through IP_3_Rs can facilitate their activity. This positive feedback underlies Ca^2+^-induced Ca^2+^ release (CICR), the regenerative process that is ubiquitously involved in the generation of diverse Ca^2+^ signals [1,6]. Serving as a co-agonist at a relatively low level, cytosolic Ca^2+^ suppresses the activity of IP_3_Rs by occupying the inhibitory site at higher concentrations [1,3,19]. This multimodal control of IP_3_Rs by IP_3_ and Ca^2+^ is central to diverse modes of intracellular Ca^2+^ signaling [1,20].

Although IP_3_R1, IP_3_R2, and IP_3_R3 share 60–80% homology at the amino acid level, they are dissimilar in sensitivity towards the primary agonists IP_3_ and Ca^2+^ and differ in their regulatory mechanisms [9,10,11,12,18]. Multiple intracellular regulators, from small molecules to proteins, have been reported to control IP_3_R activity in an isoform-specific manner and depending on intracellular context. The list of regulators includes ATP [21], cAMP [22], NADH [23], H^+^ [24], calmodulin, and several other Ca^2+^-binding proteins [18,25], as well as the IP_3_R binding proteins IRBIT and IRAG [26,27]. A variety of protein kinases and phosphatases is also involved in the regulation of IP_3_Rs [10,18,28].

**Figure 1:**
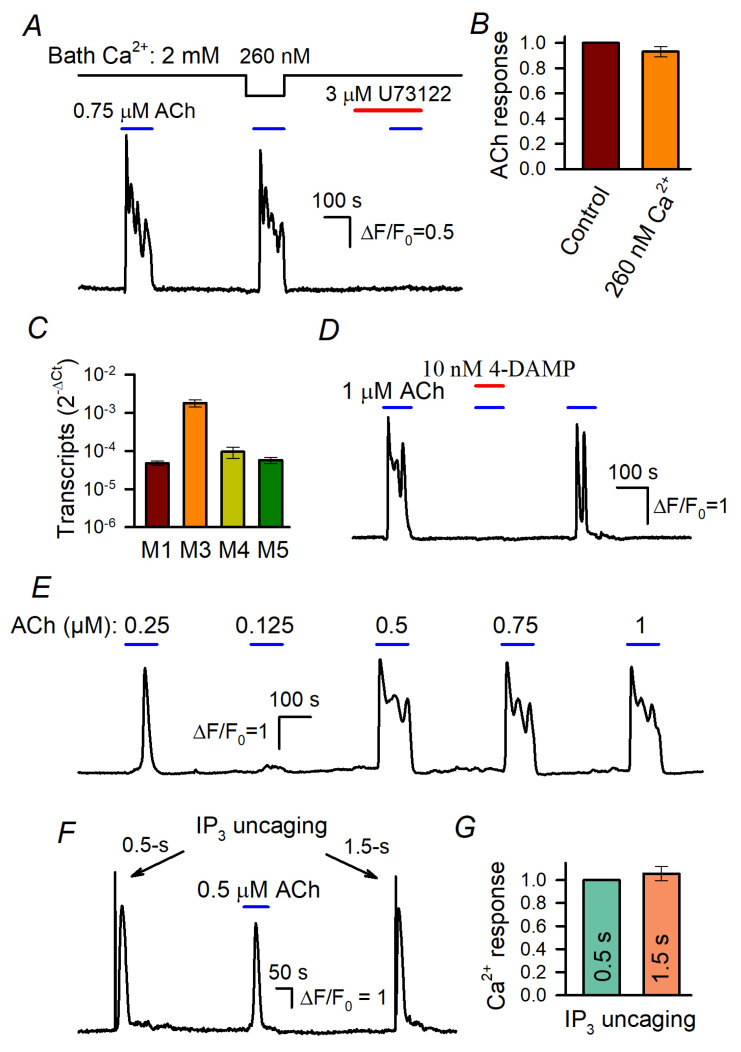
Stimulated Ca^2+^ signals in WT-HEK cells loaded with Fluo-8 4. (**A**) Evidence that Ca^2+^ release is mainly responsible for ACh-induced Ca^2+^ transients. Reduction in bath Ca^2+^ from 2 mM to 260 nM weakly affected Ca^2+^ responses to 0.75 μM ACh. The PLC inhibitor U73122 (3 μM) suppressed MSC responsivity to ACh. Here and below, the applications of compounds are indicated by the straight-line segments above the experimental trace; the data are presented as ΔF/F_0_, where ΔF = F − F_0_, F is the instant intensity of cell fluorescence, and F_0_ is the intensity of cell fluorescence obtained at the very beginning of a recording and averaged over a 20 s interval. (**B**) Summary of ACh responses at 2 mM and 260 nM Ca^2+^ in the bath. Each response at low extracellular Ca^2+^ was normalized to a response at 2 mM Ca^2+^ in the bath. The data are presented as a mean ± S.D. (81 cells). (**C**) Relative levels of muscarinic receptor transcripts in WT-HEK cells (mean ± S.D., *n* = 3) (see Supplementary Materials for details). (**D**) ACh responses were reversibly suppressed by the M3/M5 antagonist 4-DAMP at 10 nM. (**E**) Representative responses of WT-HEK cells (*n* = 256) to ACh applied at different doses, as indicated. (**F**,**G**) IP_3_ uncaging by 0.5 s and 1.5 s UV flashes elicited similar Ca^2+^ transients that were reminiscent of Ache responses. In (**G**), the data are presented as a mean ± S.D (26 cells).

Previously, we studied Ca^2+^ signaling induced by GPCR agonists, including ATP, UTP, adenosine, ACh, 5-HT, and glutamate, in cells of diverse types [29,30,31,32]. The assayed cells universally responded to Ca^2+^-mobilizing agonists in an “all-or-nothing” manner: They either were irresponsive to a particular agonist at subthreshold concentrations or generated quite similar Ca^2+^ signals at different agonist doses above the threshold (see Figure 1 below). The body of evidence suggests that being a trigger-like self-driven process, CICR finalized agonist transduction by forming cellular responses of a virtually universal shape, regardless of agonist doses [31]. Given that assayed cells express multiple IP_3_R isoforms, it remains unclear whether the “all-or-nothing” responsiveness is mediated by a specific IP_3_R subtype or whether each IP_3_R isoform can endow agonist-induced Ca^2+^ signaling in cells with this feature.

As demonstrated in previous studies, a cell line expressing a particular IP_3_R isotype represents a promising cellular model for the systematic assay of gating, regulation, pharmacology, and physiology of IP_3_R isoforms [33,34,35,36,37,38,39]. Here, we generated several such cell lines by inactivating two out of three IP_3_R genes in HEK-293 cells using the CRISPR/Cas9 approach. Different aspects of intracellular Ca^2+^ signaling in these cells were analyzed with Ca^2+^ imaging. It was particularly shown that the engineered cells responded to ACh in the “all-or-nothing” manner and that the ACh sensitivity of a given cellular subclone strongly correlated with EC_50_ for IP_3_ characteristic of an IP_3_R isoform it expressed. A mathematical model of intracellular Ca^2+^ signals induced by thapsigargin, a SERCA inhibitor, was proposed, based on which we developed an approach for estimating relative Ca^2+^ permeability of Ca^2+^ store. Altogether, our results suggest that the engineered cell lines could provide a relatively simple and effective assay of activity, regulation, and pharmacology of individual IP_3_R isoforms.

## 2. Materials and Methods

### 2.1. Cell Culture and Transfection

WT-HEK cells and their derivatives were routinely cultured in Dulbecco’s modified Eagle’s medium (DMEM) (Corning, NY, USA) containing 10% (*v*/*v*) fetal bovine serum (Cytiva, Marlborough, MA, USA) and the antibiotic gentamicin (100 μg/mL) (Corning, NY, USA) on 12-well culture plates. Cells were grown in a humidified atmosphere containing 5% CO_2_ at 37 °C.

To induce transient expression of genetically encoded Ca^2+^ sensor R-CEPIA1er with reticular location, cells were transfected with the plasmid vector pCMV R-CEPIA1er kindly provided by Masamitsu Iino (Addgene plasmid # 58216; http://n2t.net/addgene:58216, accessed on 1 October 2019; RRID:Addgene_58216) [40]. Before the day of transfection, 3–5 × 10^5^ cells were placed in the well of 12-well culture plates. For the transfection of cultured cells, the growth medium was replaced with the transfection mixture, containing 800 μL of the growth medium as well as 200 μL OptiMEM media, 2 μL P3000 Reagent, 2 μL Lipofectamine 3000 (all from Invitrogen, Waltham, MA, USA), and 2 μg pCMV R-CEPIA1er. After 24 h of incubation, the transfection mixture was replaced with the normal culture medium. The transfection was considered effective if at least 30% of the transfected cells exhibited sufficient fluorescence in the red spectral range. Next, the transfected cells were subjected to selection in the presence of antibiotic G418 (700 μg/mL) (Corning) for two weeks. The survivor cells yielded a population, which was maintained in the presence of 300 μg/mL G-418, wherein a nearly 70% cell fraction stably expressed R-CEPIA1er.

### 2.2. Ca^2+^ Imaging and IP_3_ Uncaging

Isolated cells were plated on a photometric chamber (~150 μL), which contained a disposable coverslip (Menzel-Glaser, Braunschweig, Germany) with an attached ellipsoidal resin wall. The chamber bottom was coated with Cell-Tak (Corning, NY, USA), enabling strong cell adhesion. Attached cells were loaded with Fluo-8 at room temperature (23–25 °C) by adding Fluo-8 AM (3 μM) and Pluronic^®^ F-127 (0.02%) (both from AAT Bioquest, Pleasanton, CA, USA) to the following bath solution (mM): 140 NaCl, 5 KCl, 2 CaCl_2_, 1 MgCl_2_, 10 HEPES-NaOH (pH 7.4), 10 glucose. After 30 min of incubation, the cells were rinsed several times with the bath solution and stored for 40 min to complete dye de-esterification. For IP_3_ uncaging, the cells were treated with 3 μM Fluo-8 AM + 4 μM caged-Ins(145)P3/PM (SiChem, Bremen, Germany) + 0.02% Pluronic as described above. When necessary, 2 mM CaCl_2_ in the bath were replaced with 0.5 mM EGTA + 0.4 mM CaCl_2_, thus reducing free Ca^2+^ to nearly 260 nM at 23 °C, as calculated with the Maxchelator program (http://maxchelator.stanford.edu, accessed on 1 October 2019). All used chemicals were applied with a gravity-driven perfusion system, which enabled the complete replacement of the bath solution in the photometric chamber for nearly 2 s. The used salts and buffers were from Sigma-Aldrich (St. Louis, MO, USA); the ACh and inhibitors were from Tocris Bioscience (Bristol, UK).

Experiments were carried out using an inverted fluorescent microscope Axiovert 135 equipped with an objective Plan NeoFluar 20×/0.75 (Zeiss, Oberkochen, Germany) and a digital ECCD camera LucaR (Andor Technology, Belfast, Northern Ireland). Apart from a transparent light illuminator, the microscope was equipped with a handmade system for epi-illumination via an objective. The epi-illumination was performed using a bifurcational glass fiber. One channel transmitted irradiation of computer-controlled LEDs, which provided sequential excitation of Fluo-8 or R-SEPIA1er at 480 ± 5 and 572 ± 17 nm, respectively. Their emission was collected at 535 ± 20 and 630 ± 30 nm, respectively. Serial fluorescent images were captured every second and analyzed using NIS-Elements software (version AR 5.30.01) (Nikon, Tokyo, Japan). Deviations of cytosolic and reticular Ca^2+^ in individual cells were quantified by a relative change in intensity of Fluo-8 and R-SEPIA1er fluorescence (ΔF/F_0_), respectively. Another channel was connected to a TECH-351 Advanced pulsed solid laser (680 mW) (Laser-Export, Moscow, Russia). This unit operated in a two-harmonic mode and generated not only 351 nm UV light used for Ca^2+^ uncaging but also visible light at 527 nm. The last could penetrate an emission channel through non-ideal optical filters and elicited optical artifacts during uncaging.

### 2.3. Generation of Cell Lines with Inactivated IP_3_R Genes

Two out of three IP_3_R genes in HEK-293 cells were inactivated sequentially using CRISPR/Cas9 technology. Firstly, three monoclonal cell lines were generated, HEK-∆IP3R1, HEK-∆IP3R2, and HEK-∆IP3R3, wherein *IP_3_R1*-, *IP_3_R2*-, and *IP_3_R3* were disrupted, respectively. Next, by inactivating either of two remaining IP_3_R genes in these lines, IP3R1-HEK, IP3R2-HEK, and IP3R3-HEK cells were obtained with solely IP_3_R1, IP_3_R2, or IP_3_R3, respectively, to be functional. The used methods, protocols, and controls are sufficiently detailed in the Supplementary Materials.

#### 2.3.1. Inactivation of IP_3_R1 Gene

The construct used for *IP_3_R1* inactivation was engineered on the basis of the AIO-GFP vector that provided expression of Cas9-D10A nickase fused with the enhanced green fluorescent protein (EGFP) [41]. This vector was kindly provided by Steve Jackson (Addgene plasmid # 74119; http://n2t.net/addgene:74119, accessed on 30 March, 2017; RRID: Addgene_74119). The appropriate protospacer locus was identified using IP_3_R1 mRNA sequences (GenBank NM_001099952.4; NM_001168272.2; NM_001378452.1; NM_002222.7). Target-specific sgRNAs were designed and cloned into the AIO-GFP vector as described in the Supplementary Materials. The final cAIO-GFP-sgRNA construct was verified by sequencing. WT-HEK cells were transfected with cAIO-GFP-sgRNA using Lipofectamin 3000 (Invitrogen, Waltham, MA, USA). Seventy-two hours after transfection, EGFP-expressing cells were sorted using a FACSAria SORP sorter (BD Biosciences, NJ, USA) and grown as single cells. Once a particular cell monoclone achieved a monolayer, the cells were collected, their genomic DNA was isolated, and a gene fragment containing the target site was amplified using PCR with specific primers. Each amplicon was subjected to in vitro hydrolysis using commercial nuclease Cas9 and synthesized sgRNA to reveal induced mutations in the *IP_3_R1* gene in a source clone. Finally, 4 cell clones (HEK-∆IP3R1) were found to contain necessary biallelic mutations inactivating the *IP_3_R1* gene, which also were verified by sequencing. Due to exhibiting the highest fraction (~90%) of cells responsive to ACh with Ca^2+^ transients, one clone was chosen for future experimentations.

#### 2.3.2. Inactivation of IP_3_R2 and IP_3_R3 Genes

The *IP_3_R2* and *IP_3_R3* genes were inactivated using the pGuide-it-tdTomato vector (Takara Bio, San Jose, CA, USA) that encoded nuclease Cas9 fused with the red fluorescent protein tdTomato. The appropriate protospacer locus was identified using the *IP_3_R2/IP_3_R3* mRNA sequence (GenBank NM_002223.4/NM_002224.4). Target-specific sgRNA-IP_3_R2/IP_3_R3 was designed and cloned into the pGuide-it-tdTomato vector as described in the Supplementary Materials. After verification by sequencing, the final pGuide-it-tdTomato-sgRNA-IP_3_R2/IP_3_R3 construct was transfected into WT-HEK cells. In seventy-two hours, tdTomato-positive cells were collected using a FACSAria SORP sorter and then grown as single cells. Genomic DNA was isolated from a particular monoclone, and a gene fragment containing a target site was amplified using PCR with specific primers. Each amplicon was subjected to in vitro hydrolysis using commercial nuclease Cas9 and synthesized sgRNA to reveal mutations in the *IP_3_R2*/*IP_3_R3* gene in cells of source clones. Overall, 3 HEK-∆IP3R2 and 4 HEK-∆IP3R3 clones were identified to contain inactivating indels in both alleles of the *IP_3_R2* and *IP_3_R3* genes, which were verified by sequencing. All these HEK-∆IP3R2/HEK-∆IP3R3 clones were assayed with Ca^2+^ imaging, and one exhibiting the highest fraction (~90%) of ACh-responsive cells was taken for future experimentations.

#### 2.3.3. Generation of IP3R1-HEK, IP3R2-HEK, and IP3R3-HEK Lines

Cells expressing the only *IP_3_R1* gene were generated by disrupting the *IP_3_R2* gene in HEK-∆IP3R3 cells using the strategy described above. In a variety of HEK-∆IP3R3-derived subclones, solely one cell clone, IP3R1-HEK, was eventually identified to contain proper biallelic inactivating mutations in both *IP_3_R2* and *IP_3_R3* genes.

Cells, wherein solely IP_3_R2 or IP_3_R3 was functional, were generated by inactivating the *IP_3_R3* or *IP_3_R2* gene in HEK-∆IP3R1 cells, respectively. Single-cell clones of each type, IP3R2-HEK and IP3R3-HEK, were eventually obtained (see Supplementary Materials).

### 2.4. RT-PCR and RT-qPCR

For the expression analysis, total RNA was routinely isolated from a particular cell colony (~10^6^ cells), using the Gen Elute Mammalian Total RNA Miniprep Kit (Sigma, St. Louis, MO, USA) according to the manufacturer’s protocol. Reverse transcription was performed using SuperScript IV VILO Master Mix (Thermo Fisher Scientific, Waltham, MA, USA). PCR amplification of the target sequences was performed using Phusion Hot Start II High-Fidelity DNA Polymerase (Thermo Fisher Scientific, Waltham, MA, USA) and gene-specific primers (Table S2). The primers were intron-spanning and designed to recognize transcript sequences of all known splice variants, should they be characteristic of an assayed gene. Different RNA transcription levels were quantified with the RT-qPCR approach using a real-time PCR instrument DTlight (DNA Technology, Protvino, Russia) and Maxima SYBR Green/ROX qPCR Master Mix (Thermo Fisher Scientific, Waltham, MA, USA). Amplifications were performed starting with a 3 min template denaturation step at 94 °C, followed by 45 cycles of denaturation at 94 °C for 20 s and combined primer annealing/extension at the gene specific primer temperature for 30 s (Table S2). All samples were amplified in triplicate and the mean was obtained for further calculations. Relative quantification of gene expression was calculated using the 2^–ΔCt^ method and β-Actin gene as an endogenous reference.

### 2.5. Western Blot

Expression of a particular IP_3_R isoform in the WT-, IP3R1-, IP3R2-, and IP3R3-HEK cells was verified with the Western blot approach (Figure S11). In each case, cells (~10^7^) were pelleted and directly transferred to 300 µL of 1x Laemmli buffer. Samples were incubated for 10 min at 95 °C, and each probe (20 µL) was applied on 4–15% BIS-TRIS gradient gel. Protein transfer was performed on 0.45 µM nitrocellulose membranes (BioRad Laboratories, Hercules, CA, USA) using the PowerBlotter semi-dry transfer system (BioRad Laboratories, Hercules, CA, USA). Membranes were probed with primary antibodies to IP_3_R1 (rabbit polyclonal antibodies against rat IP_3_R1 2732–2750 aa, Alomone Labs, Jerusalem Israel, Cat# ACC-019), type 2 (rabbit polyclonal antibodies against rat IP_3_R2 (2683–2696 aa, Alomone Labs, Jerusalem, Israel, Cat# ACC-116), or human IP_3_R3 (mouse monoclonal antibodies against 22–230 aa, BD Transduction Laboratories, NJ, USA, Cat# 610312). Next, probes were incubated with horseradish peroxidase-conjugated secondary antibodies Anti-Rabbit gG Peroxidase Conjugate (Sigma-Aldrich, St. Louis, MO, USA, Cat# A-9169) and Anti-Mouse IgG, IgA, and IgM Peroxidase Conjugate (IMTEC, Moscow, Russia, Cat# P-GAM Iss). Anti-actin was from United States Biological (Cat# A0760-40). Blots were imaged using iBright™ CL750 Imaging System (Thermo Fisher Scientific, Waltham, MA, USA) and enhanced chemiluminescent substrate SuperSignal™ West Pico PLUS Chemiluminescent Substrate (Thermo Fisher Scientific, Waltham, MA, Cat#34580).

## 3. Results

Reportedly, cells of the HEK-293 line endogenously express GPCRs of multiple types, including muscarinic, purinergic, chemokine, lysophospholipid, and protease receptors, many of which are coupled to the phosphoinositide cascade [42]. We therefore assayed the responsiveness of basic HEK-293 cells (WT-HEK) and their genetically modified offspring to a variety of GPCR agonists with Ca^2+^ imaging. Among agonists capable of mobilizing intracellular Ca^2+^, acetylcholine (ACh) was most effective in that it mobilized cytosolic Ca^2+^ in most (80–90%) of the assayed cells. So, cell responsiveness to ACh was predominantly analyzed in the experiments described below. WT-HEK cells that expressed all three IP_3_R isotypes (Figure S6) were subjected to gene editing using the CRISPR/Cas9 technology, and three monoclonal cell lines, each expressing a particular IP_3_R isoform, were generated (see Supplementary Materials). Here, we performed the comparative physiological analysis of Ca^2+^ signaling in WT-HEK cells as well as in cells of three lines, called IP3R1-HEK, IP3R2-HEK, and IP3R3-HEK, which functionally express solely IP_3_R1, IP_3_R2, and IP_3_R3, respectively.

### 3.1. ACh Responses of WT-HEK Cells

**Figure 2:**
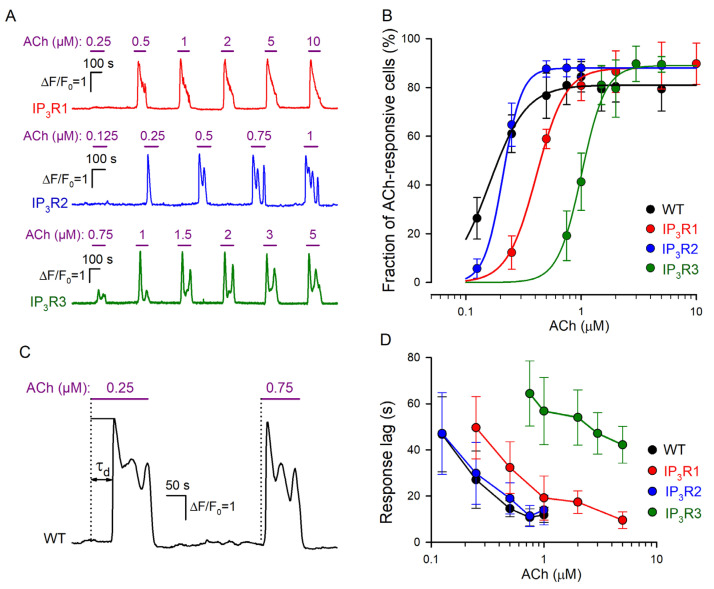
Dose dependencies of agonist responses. (**A**) Representative Ca^2+^ responses of individual cells of the IP3R1-HEK (191 cells), IP3R2-HEK (178 cells), and IP3R3-HEK (164 cells), which were serially stimulated by ACh at the indicated concentrations. (**B**) Fractions of ACh-responsive cells in WT-HEK (256 cells), IP3R1-HEK (191 cells), IP3R2-HEK (178 cells), and IP3R3-HEK (164 cells) populations. The data are presented as mean ± S.D. The straight lines correspond to the approximation of experimental data for WT-HEK-, IP3R1-HEK-, IP3R2-HEK-, and IP3R3-HEK cells with Equation (1) at the following parameters, respectively: *F*_0_ = 81, 88, 88, and 89; *C*_0_._5_ = 0.17, 0.21, 0.41, and 1.01 μM; and *n* = 2.7, 5.3, 3.4, and 4.2. (**C**) Representative Ca^2+^ transients elicited by ACh at 0.25 μM (near-threshold concentration) and 0.75 μM in the same WT-HEK cell. These ACh responses were delayed relative to the moment of agonist application by 52 s and 14 s, respectively. The characteristic time of the response delay (τ_d_) was determined as a time interval necessary for a Ca^2+^ transient to reach the half-magnitude. (**D**) Response lag versus agonist concentration (mean ± S.D.). The data were collected from 61, 56, 58, and 49 cells of the WT-HEK, IP3R1-HEK, IP3R2-HEK, and IP3R3-HEK lines, respectively.

In a particular experiment, 100–120 cells loaded with Fluo-8 were assayed simultaneously using Ca^2+^ imaging. When applied shortly, ACh elicited Ca^2+^ transients in WT-HEK (Figure 1A) and in cells of the derived lines (Figure 2A). In general, both Ca^2+^ release from intracellular Ca^2+^ store and Ca^2+^ entry mediated by a variety of Ca^2+^-permeable channels contributed to agonist-induced mobilization of cytosolic Ca^2+^. Although to our knowledge, HEK-293 cells do not express nicotinic ACh receptors, receptor-operated channels and store-operated channels (SOCs), which serve in apparently all cell types [6], contributed to the ACh responses.

It turned out that the decrease in bath Ca^2+^ from 2 mM to 260 nM affected cellular responses to ACh insignificantly (Figure 1A,B), thus indicating that IP_3_-driven Ca^2+^ release was mostly responsible for ACh-induced Ca^2+^ signals. The rationale for the abovementioned Ca^2+^ protocol was that the complete removal of bath Ca^2+^ with EGTA initiated dramatic rundown of cell responsivity during prolonged recordings. On the other hand, cells tolerated the reduction in extracellular Ca^2+^ to 260 nM, which proportionally, i.e., by the four orders of magnitude, decreased Ca^2+^ influx, in fact nullifying its contribution. In addition, the inhibition of PLC with U73122 (3 μM) rendered cells irresponsive to ACh (Figure 1A). These findings indicate that ACh transduction involved primarily muscarinic receptors that were coupled by the phosphoinositide cascade to IP_3_-driveen Ca^2+^ release rather than to Ca^2+^ entry.

The previous transcriptome analysis suggests that the M3-receptor was the predominant muscarinic isoform expressed in WT-HEK [42]. We confirmed this finding and also evaluated the M3 transcripts to be much more abundant compared to the other M-receptors (M1, M4, and M5) expressed in WT-HEK cells (Figure 1C and Figure S7). Consistently, ACh responses completely disappeared in the presence of 10 nM 4-DAMP (Figure 1C), an antagonist specific to the human M3 and M5 receptors [43].

The peculiar feature of ACh responses was their dose dependence. At concentrations below the threshold of 150–200 nM, ACh insignificantly affected intracellular Ca^2+^ in WT-HEK cells but the agonist elicited Ca^2+^ transients of similar magnitudes at a variety of higher doses (Figure 1E). This “all-or-nothing” fashion was also characteristic of agonist-induced Ca^2+^ responses in cells of several other types [29,30,31,32]. Such a step-like dose dependence should be intrinsic to agonist transduction that involves CICR, the mechanism capable of producing a large and global Ca^2+^ signal of universal shape, regardless of agonist concentrations [31]. The involvement of IP_3_R-mediated CICR in the generation of ACh responses (Figure 1E) was verified by the observation that 0.5 s and 1.5 s UV pulses elicited ACh response-like Ca^2+^ transients that were similar kinetically and by magnitude (Figure 1F,G), although the former should have uncaged a nearly three times smaller amount of IP_3_.

### 3.2. Responses of IP3R1-, IP3R2-, and IP3R3-HEK Cells to ACh

In designated experiments, we assayed IP3R1-HEK-, IP3R2-HEK-, and IP3R3-HEK cells and found that irrespective of the line, most (~90%) of them responded to brief ACh pulses with Ca^2+^ transients (Figure 2A). Similar to WT-HEK cells (Figure 1E), cells of each particular line responded to the agonist in an “all-or-nothing” manner (Figure 2A). Being associated with CICR (Figure 1F), the “all-or-nothing” responsivity of IP3R1-HEK-, IP3R2-HEK-, and IP3R3-HEK cells was consistent with the idea that each IP_3_R isoform was capable of mediating CICR [44,45]. Given the nearly step-like responsiveness of individual cells (Figure 1E and Figure 2A), their sensitivity to ACh could not be characterized by Ca^2+^ response magnitude. As an alternative dose dependence, each assayed population was evaluated by a fraction of cells responsive to the agonist at a particular concentration. After being averaged over all the experiments (*n* = 7–11) (Figure 2B, symbols), the data were fitted using the nonlinear regression approach and the Hill equation (Figure 2B, continuous lines):

(1) $$F\left(C\right)=F_{0}\frac{C^{n}}{C_{0.5}^{n}+C^{n}}$$

where *F*(*C*) is the fraction of responsive cells at the given ACh concentration *C*, *F*_0_ is the maximal fraction of ACh responsive cells, *C*_0.5_ is the EC_50_ dose, and *n* is the Hill coefficient. Based on this approximation, EC_50_ for WT-, IP3R1-, IP3R2-, and IP3R3-HEK cells were estimated as 0.16 ± 0.006, 0.21 ± 0.007, 0.41 ± 0.013, and 1.01 ± 0.05 μM, respectively. Thus, by sensitivity to ACh, the assayed cell lines were ranked as WT-HEK ≈ IP3R2-HEK > IP3R1-HEK > IP3R3-HEK.

Consistent with our previous studies of agonist-induced Ca^2+^ signaling [30,31], cells of all assayed lines responded to ACh with evident delay relative to the moment of agonist application, depending on the dose of the agonist (Figure 2C). We determined the characteristic time of the response lag (*τ*_d_) as a time interval necessary for a Ca^2+^ transient to reach the half magnitude (Figure 2C). The common feature of response lags was that *τ*_d_ markedly decreased with increasing agonist concentration (Figure 2C,D) in a cell-line-specific manner (Figure 2D). Being similarly sensitive to ACh (Figure 2B), WT-HEK and IP3R2-HEK cells showed quite similar dependencies of response lag on ACh concentration (Figure 2B). In the case of IP3R1-HEK and IP3R3-HEK cells, response lag versus ACh dose was shifted to the right (Figure 2D), consistent with lower sensitivities of both lines to ACh (Figure 2B).

Note that recently, we developed a mathematical model of agonist-induced Ca^2+^ signaling, which properly simulated cell responsivity of the “all-or-nothing” type [31]. This model predicted that for the phosphoinositide cascade with IP_3_R as the only type, its affinity to IP_3_ should be a key factor that determines the lag of Ca^2+^ responses. Note that based on their affinity to IP_3_, different IP_3_R isoforms follow the sequence IP_3_R2 > IP_3_R1 > IP_3_R3 [10,45,46,47]. It is therefore likely that only sensitivity of the particular IP_3_R isoform to IP_3_ determines the dose–response curve characteristic of the cell line wherein it is expressed (Figure 2B).

### 3.3. Thapsigargin Test of WT-, IP3R1-, IP3R2-, and IP3R3-HEK Cells

**Figure 3:**
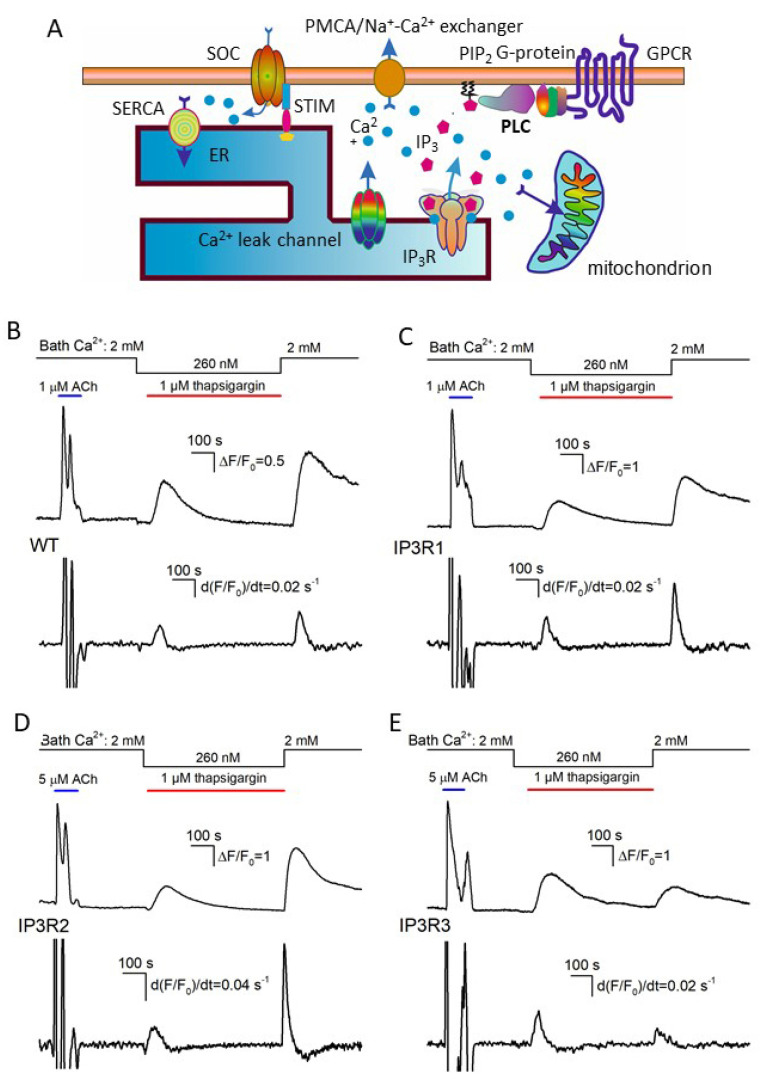
Ca^2+^ signals associated with SERCA inhibition and Ca^2+^ store depletion by thapsigargin. (**A**) Diagram showing the key contributors to intracellular Ca^2+^ signals. (**B**–**E**) Upper traces, representative Ca^2+^ transients elicited in WT-HEK cells (*n* = 149) (**B**), IP3R1-HEK (*n* = 135) (**C**), IP3R2-HEK (*n* = 101) (**D**), and IP3R3-HEK (*n* = 117) (**E**) by 1 μM ACh in control, by 1 μM thapsigargin at 260 nM Ca^2+^ in the bath, and by 2 mM bath Ca^2+^ after 600 s depletion of Ca^2+^ store. The bottom panels show derivatives $\frac{d(\frac{F}{F_{0}})}{dt}$ of the upper traces. The values of the first and second peaks were taken as measures for the rates of Ca^2+^ release (*R*_r_) and Ca^2+^ entry (*R*_e_), respectively.

Several Ca^2+^-transporting systems are universally involved in Ca^2+^ homeostasis in resting and stimulated cells [46,47]. To interpret the experiments described below, we employed a simplified model of Ca^2+^ homeostasis in assayed cells (Figure 3A). It was suggested that a level of cytosolic Ca^2+^ was mainly determined by Ca^2+^ fluxes through the plasmalemma and ER membrane, although Ca^2+^-accumulating organelles, such as mitochondria, also could shape Ca^2+^ signals. As suggested in Figure 3A, IP_3_Rs, Ca^2+^ leak channels, and Ca^2+^-ATPase SERCA were pivotal players in ER, while SOCs, the Na^+^/Ca^2+^ exchanger, and Ca^2+^-ATPase PMCA mediated Ca^2+^ fluxes through the plasma membrane.

The inhibition of SERCA with thapsigargin is conventionally used to empty Ca^2+^ store through spontaneous Ca^2+^ release, thus initiating store-operated Ca^2+^ entry (SOCE) in unstimulated cells. We employed the classical thapsigargin test to clarify whether resting activity of IP_3_Rs was a factor of Ca^2+^ leakage from ER in assayed cells. In a typical experiment, cells were initially stimulated with 1 μM ACh, and Ca^2+^ homeostasis in a given cell was considered sufficiently robust if its Ca^2+^ response to the agonist was fast and exceeded 2 in terms of ΔF/F_0_ (Figure 3B–E, upper traces). Next, cells were treated with 1 μM thapsigargin with 260 nM Ca^2+^ in the bath that nullified a contribution of Ca^2+^ entry to intracellular Ca^2+^ signals. Under these conditions, thapsigargin-elicited Ca^2+^ transients were produced by Ca^2+^ leakage from ER, which emptied Ca^2+^ store and stimulated activity of SOCs, albeit SOCE was not evident at low bath Ca^2+^. Thapsigargin was applied for 600 s, which was a sufficient interval for intracellular Ca^2+^ to return apparently to the initial level. The restoration of bath Ca^2+^ to 2 mM initiated significant SOCE associated with a marked Ca^2+^ response in the cell cytosol (Figure 3B–E; upper fluorescence trace). To quantify Ca^2+^ release and Ca^2+^ entry, Ca^2+^ traces from individual cells (Figure 3B–E; upper traces) were differentiated, and maximal rates of Ca^2+^ release (*R*_r_) and Ca^2+^ entry (*R*_e_) were determined as appropriate local maximums in the d(F/F_0_)/dt curves (Figure 3B–E; upward peaks in the bottom traces).

**Figure 4:**
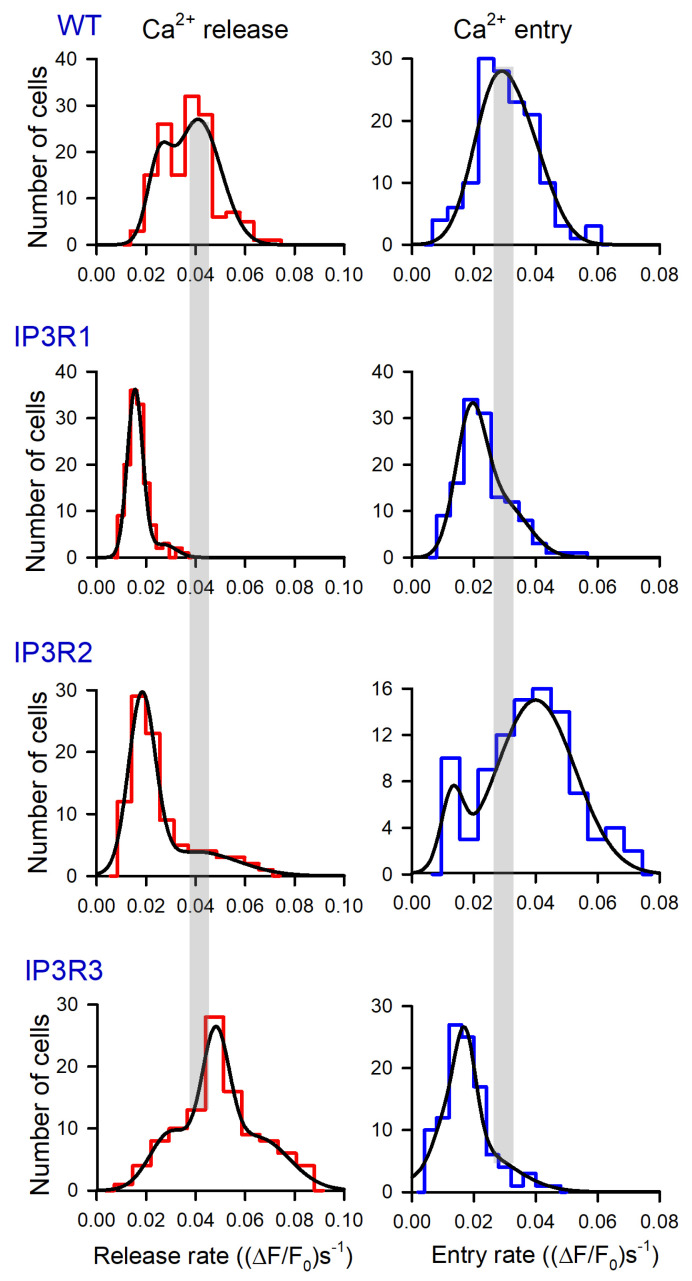
Distributions of *R*_r_ and *R*_e_ values among populations of 149 WT-HEK-, 135 IP3R1-HEK-, 101 IP3R2-HEK-, and 117 IP3R3-HEK cells, as indicated. Each experimental histogram was fitted (straight lines) using the expression: $N(r)=N_{1}e^{-(\frac{r-r_{1}}{\sigma_{1}}{)}^{2})}+N_{2}e^{-(\frac{r-r_{2}}{\sigma_{2}}{)}^{2}}+N_{3}e^{-(\frac{r-r_{3}}{\sigma_{3}}{)}^{2}}$, where *N(r)* is the number of cells exhibiting Ca^2+^ release/entry rate equal to *r*, and *N*_i_, *r*_i_, and *σ*_i_ are constants. In WT: left panel, *N*_1_ = 15, *N*_2_ = 27, *N*_3_ = 0; *r*_1_ = 2.5, *r*_2_ = 4.1; *σ*_1_ = 0.65, *σ*_2_ = 1.3. In WT: right panel, *N*_1_ = 21, *N*_2_ = 15, *N*_3_ = 0; *r*_1_ = 2.5, *r*_2_=3.7; *σ*_1_ = 0.99, *σ*_2_ = 1.1. In IP3R1: left panel, *N*_1_ = 36, *N*_2_ = 2.8, *N*_3_ = 0; *r*_1_ = 1.5, *r*_2_ = 2.7; *σ*_1_ = 0.44, *σ*_2_ = 0.73. In IP3R1: right panel, *N*_1_ = 29, *N*_2_ = 11, *N*_3_ = 0; *r*_1_ = 1.3, *r*_2_ = 4.1; *σ*_1_ = 0.71, *σ*_2_ = 1.1. In IP3R2: left panel, *N*_1_ = 28, *N*_2_ = 3.9, *N*_3_ = 0; *r*_1_ = 1.8, *r*_2_ = 4.0; *σ*_1_ = 0.76, *σ*_2_ = 2.4. In IP3R2: right panel, *N*_1_ = 6, *N*_2_ = 15, *N*_3_ = 0; *r*_1_ = 1.3, *r*_2_ = 4.1; *σ*_1_ = 0.51, *σ*_2_ = 1.8. In IP3R3: left panel, *N*_1_ = 9.3, *N*_2_ = 22, *N*_3_ = 8.5; *r*_1_ = 3.0, *r*_2_ = 4.8, *r*_3_ = 6.5; *σ*_1_ = 1.2, *σ*_2_ = 0.79, *σ*_3_ = 1.8. In IP3R3 right panel, *N*_1_ = 5, *N*_2_ = 19, *N*_3_ = 7; *r*_1_ = 1.0, *r*_2_ = 1.7, *r*_3_ = 1.9; *σ*_1_ = 0.49, *σ*_2_ = 0.5, *σ*_2_ = 1.8.

Based on these data, we generated a number of histograms to characterize distributions of *R*_r_ and *R*_e_ among robust cells of different lines. Note that satisfactory fitting for all obtained histograms could not be achieved using a single Gaussian function. Instead, it normally required a combination of two or three Gaussians (Figure 4). This implied that each particular cellular line could include two to three cell subpopulations that differed in Ca^2+^ leakage and SOCE. The level of luminal Ca^2+^, activities of Ca^2+^ leak channels and Ca^2+^ pumps, and coupling of Ca^2+^store to SOCs may have varied from cell to cell.

The experimental histograms revealed dissimilarity between WT-, IP3R1-, IP3R2-, and IP3R3-HEK cells in both thapsigargin-induced Ca^2+^ release and SOCE. In particular, the *R*_r_ distributions for WT-HEK and IP3R3-HEK cells were wider and shifted positively compared to the *R*_r_ histograms obtained for IP3R1- and IP3R2-HEK cells (Figure 4, left panels). On average, *R*_r_ in WT-, IP3R1-, IP3R2-, and IP3R3-HEK cells was 0.037 ± 0.011, 0.017 ± 0.005, 0.026 ± 0.008, and 0.049 ± 0.013 (ΔF/F_0_)s^−1^ (mean ± S.D.), respectively. This indicates that Ca^2+^ leakage in WT- and IP3R3-HEK cells was more intensive than that in IP3R1- and IP3R2-HEK cells.

For SOCE observed after the 600 s depletion of Ca^2+^ store (Figure 4, right panels), averaged *R*_r_ was 0.031 ± 0.009, 0.023 ± 0.007, 0.038 ± 0.012, and 0.018 ± 0.006 (ΔF/F_0_)s^−1^ in WT-, IP3R1-, IP3R2-, and IP3R3-HEK cells, respectively. The histograms generated for *R*_r_ also demonstrated cell-line specificity (Figure 4, right panels). Although IP3R1- and IP3R3-HEK cells were comparable to the distributions (Figure 4, right IP3R1 and IP3R3 panels) and averaged values of SOCE rates, SOC activity was higher in IP3R2-HEK cells and in WT-HEK cells (Figure 4, right panels of WT and IP3R2). These findings point at the possibility that IP_3_R2 could be functionally coupled to Ca^2+^ channels that mediate SOCE in WT- and IP3R2-HEK cells. In contrast, in H4IIE liver cells, which also express all three IP3R isoforms, primarily type 1 and, to a lesser extent, type 3, but not type 2, participated in the activation of a CRAC current associated with SOCE [48].

**Figure 5:**
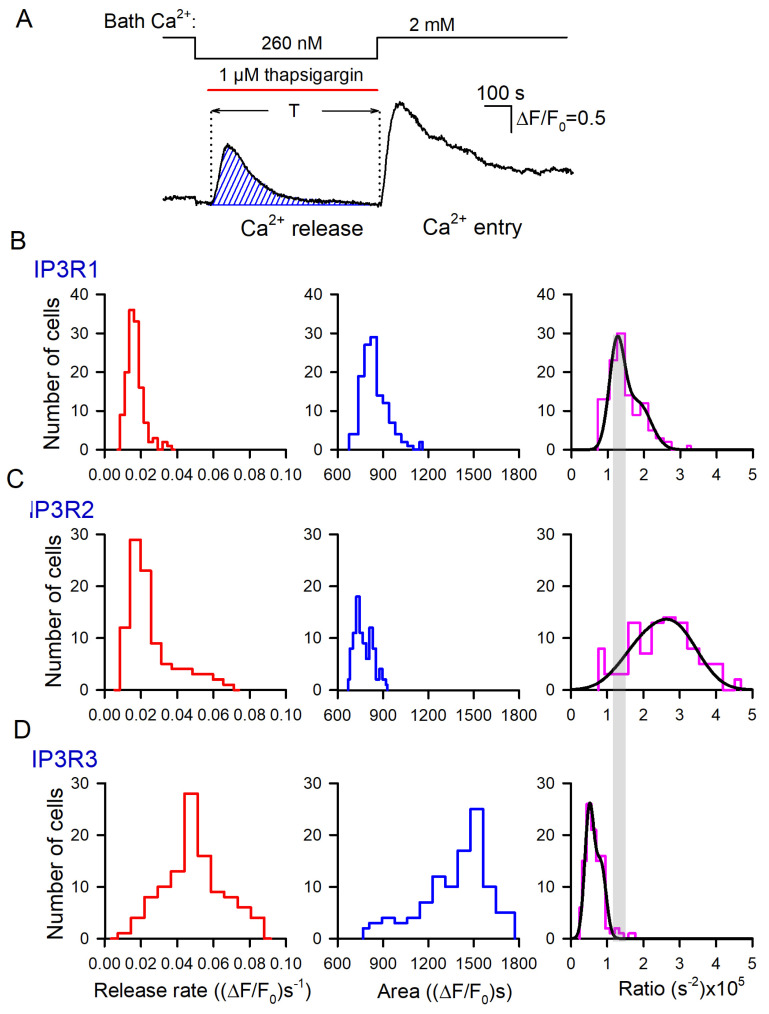
Evaluation of Ca^2+^ permeability of thapsigargin-sensitive Ca^2+^ store. (**A**) Bell-like Ca^2+^ signal produced by thapsigargin-induced Ca^2+^ release. The value of the hatched area, which corresponds to $\int_{0}^{T}Cdt$ in Equation (2), was calculated numerically. (**B**–**D**) Distributions of Ca^2+^ release rates (left panels), areas under Ca^2+^ release curves (middle panels), and their ratio (right panels) among populations of 135 IP3R1-HEK-, 101 IP3R2-HEK-, and 117 IP3R3-HEK cells, as indicated. In the right panels, each experimental histogram was fitted using the expression (straight lines) $N(r)=N_{1}e^{-(\frac{r-r_{1}}{\sigma_{1}}{)}^{2})}+N_{2}e^{-(\frac{r-r_{2}}{\sigma_{2}}{)}^{2}}$, where *N(r)* is the number of cells characterized by the rate/area ratio equal to *r*, and *N*_i_, *r*_i_, and *σ*_i_ are constants. In (**B**): *N*_1_ = 22, *N*_2_ = 12; *r*_1_ = 1.2 × 10^−5^, *r*_2_ = 1.7 × 10^−5^; *σ*_1_ = 0.29 × 10^−5^, *σ*_2_ = 0.63 × 10^−5^. In (**C**): *N*_1_ = 26, *N*_2_ = 14; *r*_1_ = 0.51 × 10^−5^, *r*_2_ = 0.83 × 10^−5^; *σ*_1_ = 0.18 × 10^−5^, *σ*_2_ = 0.21 × 10^−5^.

In resting cells, spontaneous activity of IP_3_Rs could be a factor of Ca^2+^ leakage from Ca^2+^ store [46]. Being least active at rest, IP_3_R3 should have contributed to Ca^2+^ leakage to a lesser extent compared to the other IP_3_R subtypes. However, it turned out that just Ca^2+^ store in IP3R3-HEK cells was most leaky in terms of the initial rate of thapsigargin-induced Ca^2+^ release (Figure 4, left panels). Note, however, that the Ca^2+^ release rate depended not only on Ca^2+^ permeability of the reticular membrane but also on a level of stored Ca^2+^. Thus, the Ca^2+^ release rate could not serve as an independent measure of ER permeability to Ca^2+^. To address this issue, we developed a simplified kinetics model of Ca^2+^ signals triggered by thapsigargin at low bath Ca^2+^ (Figure S13) and found the Ca^2+^ permeability *P* of the ER membrane to be proportional to the ratio (Equation (S11)):

(2) $$P\sim \frac{\frac{dC}{dt}\left(0\right)}{\int_{0}^{T}Cdt}\;$$

where *C* is the concentration of cytosolic Ca^2+^, $\frac{dC}{dt}(0)$ is the initial rate of a Ca^2+^ rise triggered by thapsigargin applied at *t* = 0, $\int_{0}^{T}Cdt$ is the area under Ca^2+^ release curve, and T is the time interval necessary for cytosolic Ca^2+^ to return to the initial level (Figure 5A).

To employ this formalism, we suggested that Fluo-8 fluorescence was far below saturation. Indeed, normally, thapsigargin-induced Ca^2+^ responses did not exceed 2 (Figure 3A), while the dynamic range of Fluo-8 should have been 20 at least [49]. Therefore, the measured parameter F/F_0_ was nearly proportional to the concentration of cytosolic Ca^2+^. For each assayed cell, we evaluated both the initial rate of Ca^2+^ release (Figure 3B–E, bottom panels) and the area under the Ca^2+^-release trace (Figure 5A, hatched area).

The appropriate histograms of $\frac{dC}{dt}(0)$ and $\int_{0}^{T}Cdt$ obtained for particular IP_3_R isoforms are presented in Figure 5B–D (left and middle panels). As illustrated, thapsigargin induced much more massive Ca^2+^ release in IP3R3-HEK cells (Figure 5D, middle panel) compared to IP3R1-HEK and IP3R2-HEK cells (Figure 5B,C, middle panels). The individual ratios (Equation (2)) were calculated, and their distributions among cells of the particular subtype were generated (Figure 5B,C, right panels). For IP3R1-HEK, IP3R2-HEK, and IP3R3-HEK cells, the averaged ratios (Equation (2)) were 1.42 ± 0.49, 2.49 ± 0.94, and 0.64 ± 0.17, respectively, and the differences between them were statistically significant (*p* < 0.05, ANOVA test). Based on these values of the ratio (2), relative Ca^2+^ permeability of the ER membrane was estimated for IP3R1-HEK, IP3R2-HEK, and IP3R3-HEK cells as 1:1.75:0.45, respectively. Thus, based on Ca^2+^ permeability of the ER, assayed cells were arranged as IP3R2-HEK > IP3R1-HEK > IP3R3-HEK. This order was rather consistent with the values of the steady-state open probabilities of IP_3_R2, IP_3_R1, and IP_3_R3 found to be ~0.3, 0.1, and <0.1, respectively, at nearly resting conditions (100 nM Ca^2+^, 1 μM IP_3_) [19]. In agreement with the previous report [39], this conformity suggested that spontaneous activity of IP_3_Rs was an essential factor of Ca^2+^ leakage from the ER. Note that the total Ca^2+^ release for 600 s (Figure 5B–D, middle panels) indicated that even being least permeable to Ca^2+^ (Figure 5B–D, right panels), Ca^2+^ store in IP3R3-HEK cells lost a larger number of Ca^2+^ ions compared to IP3R2-HEK and IP3R1-HEK cells. It was possible only if a resting level of Ca^2+^ in IP_3_-regulated Ca^2+^ store in IP3R3-HEK cells was essentially higher than one in IP3R2-HEK and IP3R1-HEK cells, provided that the ER volume was invariable among the cell lines.

### 3.4. IP3R1-, IP3R2-, and IP3R3-HEK Cells with the Ca^2+^ Sensor R-CEPIA1er

**Figure 6:**
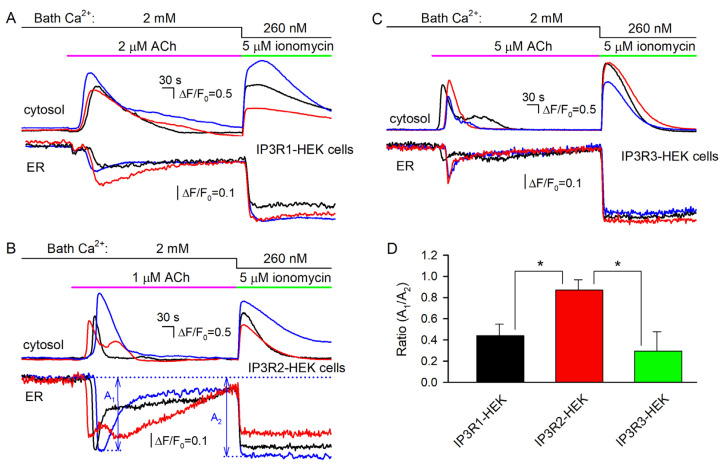
Concurrent monitoring of cytosolic and reticular Ca^2+^ in Fluo-8-loaded cells expressing the Ca^2+^ sensor R-CEPIA1er. (**A**–**C**) Representative Ca^2+^ signals in the cytosol (upper panels) and in the ER (bottom panels) of 3 individual cells assayed simultaneously, which belong to the IP3R1-HEK (**A**) (59 cells), IP3R2-HEK (**B**) (71 cells), or IP3R3-HEK (**C**) (63 cells) line. In all cases, cells were sequentially stimulated by ACh at 2 mM Ca^2+^ and by 5 μM ionomycin at 260 nM Ca^2+^ in the bath. For the particular cell line, the ACh dose was chosen to exceed EC_50_ (Figure 2B) by a factor of about 5. (**D**) Ratios of R-CEPIA1er responses on cell stimulation by ACh (A_1_) and ionomycin (A_2_), whose magnitudes were determined as shown in (**B**). The data are presented as a mean ± S.D. (*n* = 17). The asterisk indicate the statistically significant difference (ANOVA test, *p* < 0.05). There was no statistically significant difference between IP3R1-HEK and IP3R3-HEK cells.

To extend the experimental capability of the engineered lines, R-CEPIA1er, the Ca^2+^sensor with a reticular location [40], was heterologously expressed in IP3R1-, IP3R2-, and IP3R3-HEK cells. Being loaded with Fluo-8, R-CEPIA1er-positive cells allowed for simultaneous monitoring of cytosolic and reticular Ca^2+^ (Figure 6). ACh stimulated Ca^2+^ transients in the cell cytosol (Figure 5A–C, upper panels) and a synchronous drop in reticular Ca^2+^, which relaxed close to the resting level despite the agonist still being present in the bath (Figure 6A–C, bottom panels). The Ca^2+^ ionophore ionomycin (5 μM) applied at low bath Ca^2+^ (260 nM) also triggered cytosolic Ca^2+^ signals (Figure 6A–C), presumably by penetrating through plasmalemma and increasing Ca^2+^ permeability of the reticular membrane. In this case, the low steady-state level of the ionomycin response was achievable if Ca^2+^ fluxes mediated by SERCA and ionomycin were precisely balanced. The relative effects of the agonist and ionophore on reticular Ca^2+^ were quantified by the A_1_/A_2_ ratio, where magnitudes of Ca^2+^ signals elicited by ACh (A_1_) and ionomycin (A_2_) were determined as indicated in Figure 6B. As summarized in Figure 6D, ionomycin emptied Ca^2+^ store in IP3R1- and IP3R3-HEK cells to a much higher extent than ACh did (Figure 6A,C, bottom panels). In contrast, ACh and ionomycin dropped luminal Ca^2+^ in IP3R2-HEK cells to comparable levels (Figure 6B, bottom panels).

This phenomenon could be plausibly interpreted based on the recent finding that luminal Ca^2+^ inhibited activity of IP_3_Rs and related IP_3_-dependent Ca^2+^ signals in the cell cytosol [50]. This inhibitory effect was presumably mediated by the Ca^2+^-binding protein annexin A1 (ANXA1). With no ANXA1 bound, IP_3_-gated channels were sufficiently active, while at high luminal Ca^2+^ (>100 μM), ANXA1 interacted with IP_3_Rs, promoting their inhibition [50]. Given that ANXA1 is expressed in WT-HEK cells (Figure S8), the abovementioned ANXA1-mediated regulation could explain why ACh and ionomycin reduced luminal Ca^2+^ to close levels in IP3R2-HEK cells but not in IP3R1-HEK and IP3R3-HEK cells. Indeed, our findings suggest that at rest, ER permeability to Ca^2+^ was highest in IP3R2-HEK cells (Figure 5B–D, right panels), implying that a resting level of luminal Ca^2+^ should have been lower in these cells compared to IP3R1-HEK and IP3R3-HEK cells, provided that SERCA was similarly active in all cell subgroups. If so, IP_3_Rs in IP3R2-HEK cells operated in a mode characterized by a higher open probability at the same IP_3_ level [50], thus mediating higher Ca^2+^ release despite lower luminal Ca^2+^.

## 4. Discussion

The Ca^2+^ homeostasis in the ER is governed by a complex protein network, which ensures the steady-state Ca^2+^ level in the ER lumina at rest, precise Ca^2+^ release upon cell stimulation, and effective refilling of Ca^2+^ store [47,51,52,53]. At rest, the level of free Ca^2+^ in the ER lumina, which ranges between 300 and 800 μM [51], is determined by balance between passive Ca^2+^ leakage and active ER refilling by the SERCA pump [47]. The inhibition of SERCA with thapsigargin completely depletes a Ca^2+^ pool in the ER within minutes, indicating that Ca^2+^ constantly leaks from the ER [54]. Several mechanisms have been implicated in mediating Ca^2+^ leakage, including proteins from the transmembrane BAX inhibitor motif-containing (TMBIM) family, the antiapoptotic protein BCL-2, and a truncated version of SERCA pump SERCA1T [47,54]. The effective feedback mechanism preventing the overload of ER with Ca^2+^ involves TMCO1 proteins, which are capable of oligomerizing at high luminal Ca^2+^ to form transient Ca^2+^ leak channels [55]. Evidence exists that in resting cells, spontaneous activity of IP_3_Rs and ryanodine receptors (RyRs) could be responsible for a fraction of Ca^2+^ influx from the ER [47,54]. For instance, in HEK-293 cells, knockout of all three IP_3_R genes markedly slowed spontaneous Ca^2+^ release from the ER [39], indicating that resting activity of IP_3_Rs was a significant factor in Ca^2+^ leakage. On the other hand, our observations suggest that RyRs contributed negligibly to Ca^2+^ leakage from the ER in HEK-293 cells (Figure S12).

The sustained depletion of luminal Ca^2+^ is detrimental to cells, as it causes ER stress, inhibits protein synthesis, and provokes apoptosis [56,57]. Cells employ a number of mechanisms to counteract the prolonged depletion of Ca^2+^ store, including Ca^2+^-binding proteins buffering luminal Ca^2+^ [58], coupling of Ca^2+^ store depletion to activated SOCE [59], and active reloading of ER with SERCA [60]. The core mechanism of SOCE involves stromal interaction molecules (STIMs), basically STIM1 but also STIM2, which serve as sensors of ER Ca^2+^ and SOCE regulators [36]. Being initiated by Ca^2+^ store depletion, the dissociation of Ca^2+^ from STIM1 proteins causes their oligomerization and relocation to the specialized membrane contact sites between the plasma membrane and the ER, called the ER–PM junction. In this junction, the cytosolic domains in STIM1 oligomers have an unfurling and elongated conformation necessary to capture Orai channels located in the plasmalemma and enable their opening [36].

In cells of diverse types, IP_3_Rs represents the main conduit for stimulus-dependent Ca^2+^ release [3,9,46], and therefore they should be coupled to SOCs, at least functionally. The functional interaction was indeed demonstrated for IP_3_Rs and TRPC channels involved in SOCE [56,57,58,61,62] as well as between IP_3_Rs and ORAI1 [59,60]. Moreover, evidence exists that STIM proteins can directly interact with IP_3_Rs [36,63]. The recent findings point out that STIM1 forms a complex predominantly with activated IP_3_R [63]. Indeed, being a trigger of STIM1 oligomerization, a transient fall in intraluminal Ca^2+^ initiated by IP_3_ should be most pronounced just in the close vicinity of open IP_3_R.

The cell lines expressing merely one IP_3_R subtype represent a promising cellular model for the systematic assay of gating, regulation, pharmacology, and physiology of individual IP_3_R isoforms. The first vertebrate cellular model suitable for functional analysis of individual IP_3_R isotypes in the same cellular background was established based on chicken lymphoma-derived DT40 cells, wherein all three IP_3_R genes were disrupted (DT40-TKO cells) [33]. The heterologous expression of mammalian IP_3_Rs in DT40-TKO cells provided a deep insight into the functionality of individual IP_3_R isotypes. It was particularly demonstrated that constitutive Ca^2+^ release through IP_3_R to mitochondria is required for mitochondrial respiration and maintenance of normal cell bioenergetics [34]. It turned out that IP_3_R2 delivered Ca^2+^ to mitochondria most effectively, although each IP_3_R isotype can support local contact sites of ER and mitochondria [38]. The permeabilized cells expressing a particular IP_3_R subunit were employed to assay Ca^2+^ release stimulated by IP_3_ and its synthetic analogs. The dose–response curves generated for all IP_3_R subtypes yielded the first structure–activity relationships for the key IP_3_ analogues [35]. Reportedly, IP_3_-induced Ca^2+^ release was effective only if each IP_3_R monomer within the tetramer was occupied by IP_3_ [36].

Apart from DT40-TKO cells, IP_3_R-defficient HEK-293 and HeLa cells have also been generated by using CRISPR/Cas9 genome editing [36,37,39]. Being employed as a heterologous system for systematic expression and assay of mammalian IP_3_Rs, these model lines facilitated the acquisition of a number of interesting findings. It was particularly reported that upon IP_3_ uncaging, all three IP_3_R subtypes were capable of mediating Ca^2+^ puffs, relatively small and localized Ca^2+^ transients. The pathological mutations associated with dysfunction of IP_3_R1 disrupted IP_3_ binding, IP_3_-mediated gating, and its regulation by IP_3_R-modulatory proteins [37]. Being significantly reduced in HEK293-TKO cells, Ca^2+^ leakage from the ER and its refilling were rescued by overexpression of recombinant IP_3_R1 or IP_3_R3 [39].

In the present work, we developed our own cell lines suitable for the analysis of a role of individual IP_3_R isotypes in agonist-induced Ca^2+^ signaling. By inactivating two out of three IP_3_R genes in HEK-293 cells with CRISPS/Cas9 technology and employing cell selection methods, we generated three monoclonal cell lines, IP3R1-HEK, IP3R2-HEK, and IP3R3-HEK, with IP_3_R1, IP_3_R2, and IP_3_R3 being solely functional, respectively. The functional consequence of this meddling in the natural pattern of IP_3_R expression was evaluated by studying certain aspects of Ca^2+^ signaling in these genetically modified cells.

In each line, IP3R1-HEK, IP3R2-HEK, or IP3R3-HEK, cells responded to ACh with Ca^2+^ mobilization and exhibited “all-or-nothing” responsivity to the agonist (Figure 2), as was the case with WT-HEK cells (Figure 1D). Given this feature, a dose dependence of cell responses was characterized at the population level by the number of cells responsive to ACh at a particular concentration. Based on the EC_50_ doses obtained from these dose-response relationships (Figure 2B), ACh sensitivities of the genetically modified cells ranked as IP3R2-HEK > IP3R1-HEK > IP3R3-HEK. Response lags basically obeyed the same sequence (Figure 2C,D).

It should be noted that genome editing with RNA-guided nucleases, such as Cas9, can entail off-target DNA cleavage [61,62]. As an additional control, we compared assayed cells based on the expression of muscarinic receptors and several downstream signaling proteins that could be involved in ACh transduction, including G_q_- and G_i1-3_-proteins, which are known to couple a variety of GPCRs to G-protein-regulated PLCβ1-4 [56]. The relative expression levels were assessed using RT-qPCR (see Supplementary Materials).

In mammals, five genes encode muscarinic receptors of the M1–M5 subtypes. We identified M1, M3, M4, and M5 transcripts in WT-HEK cells, while M2 transcripts were undetectable (Figure S7A). Among them, the M3 isotype was dominant at the transcript level (Figure S7B). Consistently, physiological evidence validated the central role of the M3 receptor in mediating ACh-induced Ca^2+^ mobilization (Figure 1D). Although expression of the identified M receptors somewhat varied from line to line (Figure S9A), M3 transcript levels were statistically indistinguishable (Figure S9A, M3 panel). It thus appears that distinct sensitivities of IP3R1-, IP3R2-, and IP3R3-HEK cells to ACh (Figure 2B) were determined by a mechanism downstream of the M receptors.

Given that the M3 receptor primarily couples to the G_q_ protein [57], the distinct sensitivities of IP3R1-HEK, IP3R2-HEK, and IP3R3-HEK cells to ACh may be attributed to lineage-specific expression of G_q_. It was found that the IP3R1-HEK/IP3R2-HEK and IP3R2-HEK/IP3R3-HEK pairs exhibited statistically indistinguishable levels of G_q_ expression (Figure S9B, G_q_ panel). On the other hand, the level of G_q_ transcripts in IP3R3-HEK cells was ~50% lower than in IP3R1-HEK cells (Figure S9B, G_q_ panel). These data revealed no correlation between a level of G_q_ expression in a particular cell line (Figure S9B, G_q_ panel) and its responsiveness to ACh (Figure 2B). In addition, levels of G_i1-3_ transcripts in all assayed lines were statistically indistinguishable (Figure S9B, G_i_ panels). Hence, the different sensitivities of IP3R1-HEK, IP3R2-HEK, and IP3R3-HEK cells to ACh (Figure 2B) did not seem correlated with differences in their expression of G proteins (Figure S9B), which coupled M receptors to PLC.

Although expression of PLCβ1–4 was more scattered among cell populations, statistically significant deviations exhibited solely PLCβ3 transcripts (Figure S6C). Given, however, that compared to PLCβ1 and PLCβ2 the level of PLCβ3 transcripts was lower by a factor of 3–10 (Figure S8C), this PLC isoform was presumably a minor contributor to ACh signaling (Figure 2). In summary, the abovementioned results (Figures S7 and S8) indicate that the IP_3_R gene editing has had insignificant impact on the expression of proteins potentially crucial to ACh transduction.

Previously, we developed a mathematical model of agonist transduction that included the phosphoinositide signaling cascade and IP_3_-driven Ca^2+^ release through the only type of IP_3_Rs [31]. This model properly simulated the characteristic features of agonist-induced Ca^2+^ signaling, such as the “all-or-nothing” responsivity of cells (Figure 2A) and the dose dependence of the response lag (Figure 2D). In line with this model, a threshold agonist concentration in a step-like dose dependence of Ca^2+^ responses (Figure 4 in [31]) was determined by both a rate of IP_3_ production stimulated by an agonist and the affinity of IP_3_ binding to IP_3_Rs. Note that the expression analysis (Figure S9) suggests that the efficacy of the ACh transduction pathway, i.e., muscarinic receptor–G protein–PLC–IP_3_ production, was likely similar or nearly identical in IP3R1-, IP3R2-, and IP3R3-HEK cells. On the other hand, based on the revealed levels of IP_3_R transcripts (Figure S10), these cells were ranked as IP3R3-HEK > IP3R2-HEK > IP3R3-HEK, although based on ACh responsivity, they were ordered as IP3R2-HEK > IP3R1-HEK > IP3R3-HEK (Figure 2B). The last sequence aligned well with the affinities of the corresponding IP_3_Rs to IP_3_, which followed the order IP_3_R2 > IP_3_R1 > IP_3_R3 [7,11,58,59,60]. It thus appears that the IP_3_ affinity of an IP_3_R isoform operating in a particular cell line was a decisive factor that determined sensitivity of the assayed cells to ACh.

## 5. Conclusions

In this study, we generated our own monoclonal cell lines, IP3R1-HEK, IP3R2-HEK, and IP3R3-HEK, for further analysis of the role of individual IP_3_R isotypes in mediating various aspects of agonist-induced Ca^2+^ signaling. For instance, the IP3R3-HEK line could provide sufficient insight into why type II taste cells exclusively involve IP_3_R3 in sweet, bitter, and umami transduction [16]. We utilized the generated cellular models to verify several important findings reported earlier. Specifically, our data confirmed that each IP_3_R isoform could mediate the CICR process and contribute to Ca^2+^ leakage from Ca^2+^ store at rest. Furthermore, we developed a mathematical model, which allowed for the relative Ca^2+^ permeability of Ca^2+^ store to be estimated based on Ca^2+^ signals induced by thapsigargin in the cell cytosol. With this approach, relative Ca^2+^ permeabilities of Ca^2+^ store in IP3R1-HEK, IP3R2-HEK, and IP3R3-HEK cells were evaluated to be 1:1.75:0.45.

The engineered cells responded to ACh in strong correlation with the IP_3_ sensitivity of an IP_3_R isoform they expressed. Based on this correlation, any alteration in responsivity of the particular cell line, induced by a pharmacological agent targeting IP_3_Rs, could be attributed to a change in activity of the related IP_3_R isoform. We therefore anticipate that the developed cell lines, in combination with genetically encoded sensors (Figure 6), could provide a relatively straightforward and efficient means for assaying the activity, regulation, and pharmacology of individual IP_3_R isoforms.

## Data Availability

The original contributions presented in the study are included in the article/Supplementary Materials, further inquiries can be directed to the corresponding author/s.

## Supplementary Materials

The following supporting information can be downloaded at: https://www.mdpi.com/article/10.3390/cells13070562/s1, Figure S1: Fragments of the sense and antisense sequences (374-441 bp) of the IP3R1 gene; Figure S2: Confirmation of biallelic mutations in cells transfected with the cAIO-GFP-sgRNA vector; Figure S3: Biallelic mutations in the IP3R1 gene revealed in the monoclone 12; Figure S4: Fragment (414-505 nucleotides) of the IP3R2 gene containing the protospacers (blue) and PAM (in red); Figure S5: Fragment (448-478 nucleotides) of the IP3R3 gene containing the protospacers (blue) and PAM (red); Figure S6: Expression of IP3Rs in WT-HEK cells; Figure S7: Expression of muscarinic receptors in WT-HEK cells; Figure S8: Representative RT-PCR analysis (n=3) of expression of human annexin A1 in WT-HEK cells; Figure S9: Expression of signaling proteins in cells of different lines; Figure S10: Expression of IP3Rs in cells of different lines; Figure S11: Western blots from WT-, IP3R1-, IP3R2-, and IP3R3-HEK cells; Figure S12: Ca^2+^ signals associated with SERCA inhibition and Ca2+ store depletion by thapsigargin; Figure S13: Kinetics model of Ca^2+^ homeostasis in a unstimulated cell; Table S1: Mutations of the targeted IP3R gene in cells of the HEK-IP3R1, HEK-IP3R2, and HEK-IP3R3 lines; Table S2: Primer sequences. [36,63,64,65,66,67,68]
