# Supplementary material for: Agonist-Induced Ca2+ Signaling in HEK-293-Derived Cells Expressing a Single IP3 Receptor Isoform
Source: Cells. 2024 Mar 22;13(7):562. doi: 10.3390/cells13070562 (PMC11011116; doi:10.3390/cells13070562)
Supplement: Supplementary file 1 [file cells-13-00562-s001.zip › cells-2626657-supplementary.pdf]

SUPPLEMENTARY MATERIALS

I. Generation of cell lines with inactivated IP_3R genes

Inactivation of two out of three IP_3Rs in HEK-293 cells was performed using CRISPR/Cas9 technology in a sequential manner. As a first step, monoclonal lines with one out of three IP3R inactivated genes were generated, namely, HEK-293/ΔIP3R1, HEK-293/ΔIP3R2 and HEK-293/ΔIP3R3. Next, in each monoclonal line, another *IP_3R* gene was inactivated, thus resulting in a cell line with only one functional IP_3R isoform.

I.1. Inactivation of the IP_3R1gene.

Inactivation of the *IP_3R1* gene was performed using AIO-GFP vector encoding Cas9-D10A nickase fused with the enhanced green fluorescent protein (EGFP) (Chiang et al., 2016). The vector was kindly provided by Steve Jackson (Addgene plasmid # 74119; http://n2t.net/addgene: RRID: Addgene_74119). The optimal protospacer locus was identified using mRNA IP3R1 published sequences (GenBank NM_001099952.4; NM_001168272.2; NM_001378452.1; NM_002222.7); coding DNA strand sequence 5'-AAATGGATTTATTAGCACCT and antisense DNA strand sequence 5'-AACAAATGTCTCCAATATGT were targeted on all transcripts and chosen for editing (Fig.1S).

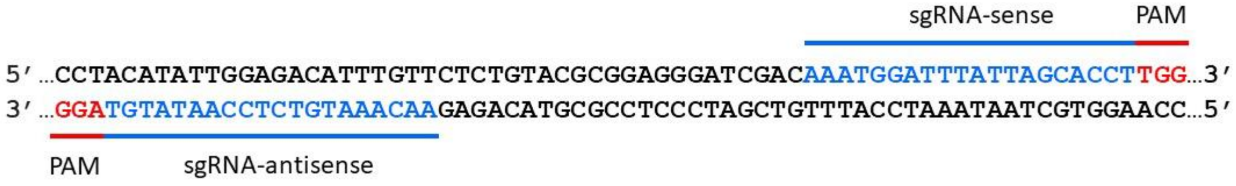

Figure 1S. Fragments of the sense and antisense sequences (374-441 bp) of the *IP3R1* gene (exon 3, chromosome 3, NC_000003.12) containing the protospacers (shown in blue) and protospacer adjacent motif (PAM) (red).

Target-specific sgRNA sequences were cloned into AIO-GFP Vector for simultaneous expression with Cas9-D10A-EGFP. Spacer sequences were created by annealing the pairs of complimentary oligonucleotides carrying overhangs for cloning on 5’-ends: 5'-accgAAATGGATTTATTAGCACCT-3' and 5'- aaacAGGTGCTAATAAATCCATTT-3', 5'-accgAACAAATGTCTCCAATATGT-3' and 5'-aaacACATATTGGAGACATTTGTT-3'. Oligonucleotides pairs were annealed, phosphorylated and then ligated with linearized dephosphorylated AIO-GFP vector one after another. First, coding strand spacer duplex was assembled with the vector linearized at BbsI restriction site. Ligation mix was transformed into Stellar Competent Cells (Takara Bio, USA) according to the user manual protocol. Colonies containing vector with correct insert were screened by colony PCR using ScreenMix-HS (UDG)

(Evrogen, Russia) master mix using insert-specific forward primer 5'-ACCGAACAAATGTCTCCAATATGT-3' and vector-specific reverse primer 5'-CTTGATGTACTGCCAAGTGGGC-3'. Final constructs were confirmed by sequencing. Then, vector containing sense strand spacer was linearized at BsaI (Eco31I) restriction site and ligated with antisense strand spacer duplex. Ligation mix was transformed into Stellar Competent Cells (Takara Bio, USA) according to user manual protocol. Colonies containing vector with correct insert were identified by PCR using ScreenMix-HS (UDG) (Evrogen, Russia) master mix and insert-specific forward primer 5'- ACCGAAATGGATTTATTAGCACCT-3' and vector-specific reverse primer 5'- CTTGATGTACTGCCAAGTGGGC-3'. Final constructs were confirmed by sequencing.

Two pairs of complementary DNA oligonucleotides were synthesized by Evrogen (Russia), and cloned into AIO-GFP vector. The following pairs of oligonucleotides were used to produce sense and antisense sgRNAs, respectively: 5'-accgAAATGGATTTATTAGCACCT-3' and 5'- aaacAGGTGCTAATAAATCCATTT-3', 5'-accgAACAAATGTCTCCAATATGT-3' and 5'-aaacACATATTGGAGACATTTGTT-3'. DNA oligonucleotides were individually phosphorylated using T4-polynucleotidekinase Anza™ T4 PNK kit (Invitrogen, USA) following the manufacturer's recommendations. To create oligonucleotide duplexes, the complementary DNA oligonucleotides (50 nM each) were mixed in 1:1 ratio, heated to 95°C for 2 min, and then gradually cooled to RT for 40 min. Each of the resulting DNA oligonucleotide duplexes had forward accg and reverse aaac 5' overhangs for further direct cloning into the backborn vector at BbsI and BsaI restriction sites. First, the antisense duplex was ligated into BbsI-digested vector plasmid using Ligation Mix (Takara Bio, USA). Then, the sense duplex was ligated into resulted vector linearized with BsaI enzyme. Stellar Competent Cells (Takara Bio, USA) were used for transformation and further production of the cAIO-GFP-sgRNA/IP$_3$R1 plasmid. The presence of the insertions in the final construct was confirmed by Sanger sequencing (Evrogen, Russia).

The cAIO-GFP-sgRNA/IP$_3$R1 construct was transfected into WT-HEK cells using Lipofectamin 3000 (Invitrogen, USA) following manufacturer's protocol. Seventy-two hours after transfection, EGFP-expressing cells were sorted using a FACSAria SORP sorter (BD Biosciences, USA) and grown as single cells in 96-well plates up to 50-70% confluency. Next, monoclonal colonies of cells were sequentially transferred into bigger wells of 24-, 12- and 6-well plates. Once a particular monoclone achieved the monolayer in a 6-well plate, cells were collected, and their genomic DNA was isolated using Quick-DNA Miniprep Plus Kit (Zymo Research, USA). The locus targeted by sgRNA (Fig.1S) was PCR amplified using Phusion Hot

Start II High-Fidelity DNA Polymerase (Thermo Fisher Scientific, Waltham, USA) and the primers, 5'-CATTCCCTGAAGCAAATTGAATATG-3' and 5'-AGTGGAATAAAGGCACTCTC-3'.

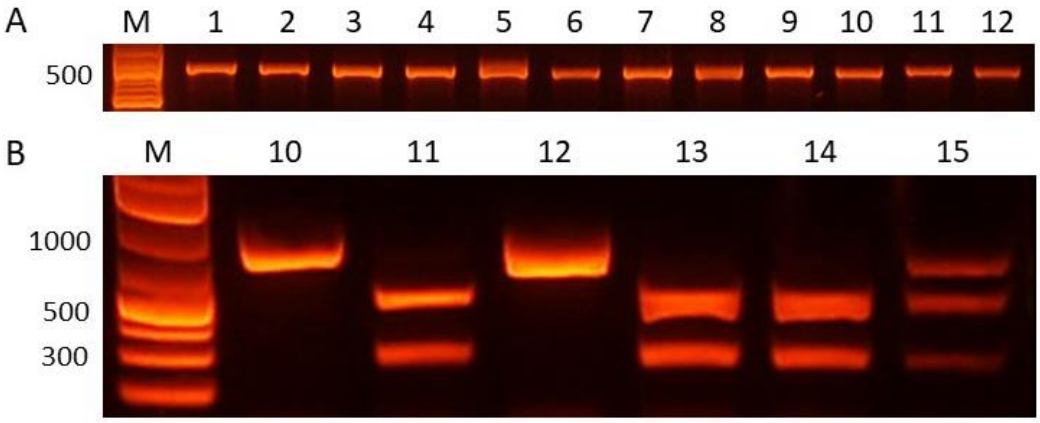

Figure 2S. Confirmation of biallelic mutations in cells transfected with the cAIO-GFP-sgRNA vector. (A) Amplicons of expected size (769 bp) validate the presence of the *IP3R1* mutation cite in genomic DNA from individual cellular clones 1-12. The similar amplicons were obtained for monoclones 13-15. (B) Products of Cas9/sgRNA mediated ***in vitro*** hydrolysis of amplicones (A) from individual cellular monoclones 10-15. Migration of DNA fragments was monitored in 1.2% agarose gel stained with EtBr; GeneRuler 100 bp Plus DNA Ladder (Thermo Fisher Scientific, Waltham, USA) molecular markers (M) were used.

Each amplicon was subjected to ***in vitro*** hydrolysis using commercial nuclease Cas9 and synthesized sgRNA to reveal the presence of mutations in the targeted *IP$_3$R1* gene. As sgRNA would only target the wild type sequence without insertion/deletion, in case of no mutations on both alleles, the amplified DNA fragment would be completely digested and present as two fragments on the agarose gel. In case of mutation present on one allele, only amplicons from the wild type allele would be digested, so the product of hydrolysis would migrate as three bands. In case of biallelic mutants, neither allele would be cleaved, resulting in the single band of 769 bp (Fig.2SB).

In total, 98 monoclonal colonies were obtained, and each one underwent the ***in vitro*** Cas9-sgRNA assay. In 57% of the tested monoclones, the *IP$_3$R1* gene was not affected, monoallelic mutation were found in 38% of the colonies, while 5 monoclones (5%) contained biallelic mutations. Fig.2SB exemplifies results of the mutation analysis of six out of the first 15 cellular monoclones. As shown, the *IP$_3$R1* gene remained unaltered in the monoclones 1st-9th , 11th, 13th, and 14th monoclones, biallelic mutation occurred in the monoclones 10th and 12th, while a monoallelic mutation of the *IP$_3$R1* gene was only found in the monoclone 15. The biallelic mutations identified in monoclones 10th and 12th were confirmed by sequencing. Monoclone 12 had a 7 nucleotide insertion on both alleles shifting the open reading frame with a stop-codon

appeared after the 28th amino acid (Fig.3S). As exhibiting the highest fraction (89%) of cells responsive to ACh with Ca^{2+} transients, the 12th monoclone named HEK-ΔIP3R1 was chosen for future experiments.

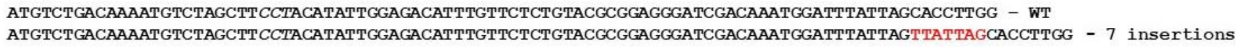

***Figure 3S*.** Biallelic mutations in the *IP3R1* gene revealed in the monoclone 12. The upper sequence represents the 351-441 nucleotides of the wild type IP3R1, the bottom sequence is the mutated locus.

I.2. Inactivation of the IP3R2 gene.

Inactivation of the *IP₃R2* gene was performed using the Guide-It CRISPR/Cas9 System (Takara Bio, USA) according to user manual protocol. Optimal protospacer locus was identified using mRNA IP_3R2 published sequence (GenBank NM_002223.4) and sequence 5'-GGACATCGTGTCCCTGTACG was targeted for editing (Fig.4SA). Target-specific sgRNA sequence was cloned into pGuide-it-tdTomato Vector for simultaneous expression with Cas9 protein as well as fluorescent protein tdTomato. Spacer sequence was created by annealing a pair of complimentary oligonucleotides carrying overhangs for cloning on 5'-ends: 5'-ccggGGACATCGTGTCCCTGTACG-3' and 5'-aaacCGTACAGGGACACGATGTCC-3'. Expression vector assembly was performed according to Guide-it CRISPR/Cas9 Systems User Manual.

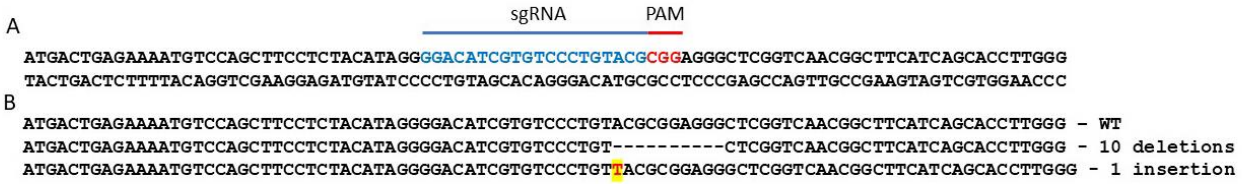

Figure 4S. (A) Fragment (414-505 nucleotides) of the *IP₃R2* gene containing the protospacers (blue) and PAM (in red). (B) Biallelic mutations of the *IP₃R2* gene found in the clone 7: one allele contains 10 deletions, while another has 1 insertion (red).

Ligated plasmids were transformed into Stellar Competent Cells (Takara Bio, USA) according to user manual protocol. Colonies containing vector with correct insert were screened by colony PCR using ScreenMix-HS (UDG) (Evrogen, Russia) master mix using insert-specific forward primer 5'-CCGGGGACATCGTGTCCCTGTACG-3' and vector-specific reverse primer 5'- AAAAGCACCGACTCGGTGCC-3'. Final constructs were confirmed by sequencing.

The pGuide-it-tdTomato-sgRNA/IP3R2 construct was transfected into WT-HEK cells using Lipofectamin 3000 (Invitrogen, USA). Seventy-two hours after transfection, tdTomato -expressing cells were sorted using a FACSAria SORP sorter (BD Biosciences, USA) and grown

as single cells in 96-well plates up to 50-70% confluency. Monoclonal colonies of cells were then sequentially transferred into bigger wells of 24-, 12- and 6-well plates. After cells reached a monolayer in a 6-well plate, genomic DNA from each monoclone was isolated by Quick-DNA Miniprep Plus Kit (Zymo Research USA), and the targeted locus was PCR amplified using primers 5'-CATTCCCTGAAGCAAATTGAATATG-3' and 5'-AGTGGAATAAAGGCACTCTC-3'. Similar to experiments that were aimed at inactivation of the *IP3R1* gene (Fig.2S), obtained amplicons were subjected to ***in vitro*** cleavage with Cas9 to reveal clones with the mutated *IP$_3$R2* gene. Overall, 54 monoclones were analyzed with this method, and biallelic mutations identified in the clones 7 and 24, which were confirmed by sequencing (Fig.4SB). It was particularly found that one allele in the clone 7 carried a 10bp deletion, shifting the open reading frame and originating the stop-codon after the 25th AA. The second allele carried an insertion of one nucleotide, originating a stop-codon after the 34th AA. As exhibiting a higher fraction of ACh responsive cells, the clone 7 was chosen for future experiments, being referred to as HEK-ΔIP3R2.

I.3. Inactivation of the IP3R3 gene.

Modification of IP3R3 gene was performed using the Guide-It CRISPR/Cas9 System (Takara Bio, USA) according to user manual protocol. Optimal protospacer locus was identified using mRNA IP3R3 published sequence (GenBank NM_002224.4) and sequence 5'-ATGTCCAGCTTTCTTCACAT was targeted for editing (Fig.5SA).

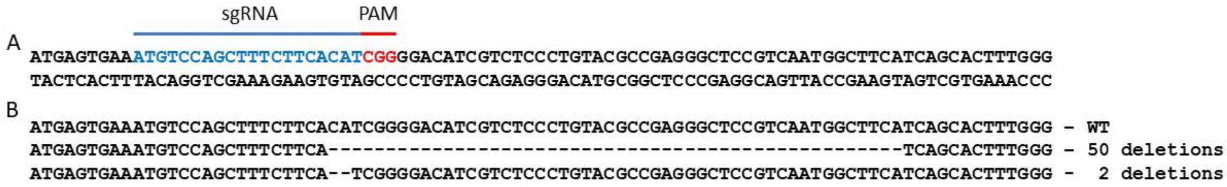

Figure 5S. (A) Fragment (448-478 nucleotides) of the *IP$_3$R3* gene containing the protospacers (blue) and PAM (red). (B) Biallelic mutations of the *IP$_3$R2* gene found in the clone 8: one allele contains 50 deletions, while another has 2 deletions.

Target-specific sgRNA sequence was cloned into pGuide-it-tdTomato Vector for simultaneous expression with Cas9 protein as well as fluorescent protein tdTomato. Spacer sequence was created by annealing a pair of complimentary oligonucleotides carrying overhangs for cloning on 5’-ends: 5'-ccggATGTCCAGCTTTCTTCACAT-3' and 5'-aaacATGTGAAGAAAGCTGGACAT-3'. Expression vector assembly was performed according to Guide-it CRISPR/Cas9 Systems User Manual. Ligated plasmids were transformed into Stellar Competent Cells (Takara Bio, USA) according to user manual protocol. Colonies containing vector with correct insert were identified by PCR using ScreenMix-HS (UDG) (Evrogen, Russia) master mix using insert-specific forward primer 5'-

CCGGATGTCCAGCTTTCTTCACAT-3' and vector-specific reverse primer 5'-AAAAGCACCGACTCGGTGCC-3'. Final constructs were confirmed by sequencing.

WT-HEK cells were transfected with the pGuide-it-tdTomato-sgRNA/IP3R3 construct using Lipofectamin 3000 (Invitrogen, USA). Seventy-two hours after transfection, tdTomato -expressing cells were sorted using a FACSAria SORP sorter (BD Biosciences, USA) and grown as single cells in 96-well plates. The growth and testing of monoclonal colonies were performed as described in the section ***I.2*** for the IP_3R2 gene. Among 20 cell monoclones, which were examined for the disruption of the *IP3R3* gene, biallelic mutations were identified by the ***in vitro*** Cas9-cleavage in the clones 2 and 8. As well-responsive to ACh, the clone 8 was chosen for future experiments, and mutations inactivating IP_3R3 were validated by sequencing (Fig.5SB). As was found, one allele contained 50 deletions, which shifted the open reading frame and originating the stop-codon after the 16th AA. Another allele contained two deletions originating a stope-codon after the 32th AA (Fig.5SB). This cellular clone was named as HEK-ΔIP3R3.

I.4. Generation of HEK-IP3R1, HEK-IP3R2 and HEK-IP3R3 lines.

The cell line HEK-IP3R1 with solely IP_3R1 being functional was produced by disrupting IP_3R2 gene in HEK-ΔIP3R3 cells using pGuide-it-tdTomato Vector/IP3R2 and methodology described above in the paragraph I.2. Overall, 25 cellular monoclones were examined. Among them, one colony exhibited unaffected IP3R1 gene and biallelic disrupting mutations of the *IP_3R2* and I*P_3R3* genes. These findings were validated by DNA Sanger sequencing that indicated the appearance of stop-codons after the 17th AA in one allele and after 31th AA in another (Table 1S).

HEK-IP3R2 cells were generated by inactivating the IP3R3 gene in HEK-ΔIP3R1 cells with the pGuide-it-tdTomato Vector/IP3R3 construct. Appropriate HEK-IP3R2 cells were found among 15 tested monoclones derived from HEK-ΔIP3R3. The associated biallelic mutations of the IP3R3 gene resulted in the stop-codons after the 32th AA in both alleles (Table 1S).

HEK-IP3R3 cells were generated by inactivating the IP3R1 gene in HEK-ΔIP3R2 cells using the cAIO-GFP-sgRNA/IP_3R1 construct. Among 20 tested positive HEK-ΔIP3R2 monoclones, one contained the desired biallelic mutations of the IP3R1 gene resulted in stop-codons after 14th and 70th AA (Table 1S).

***Table 1S*.** Mutations of the targeted IP3R gene in cells of the HEK-IP3R1, HEK-IP3R2, and HEK-IP3R3 lines*

Cell line	Targeted gene	5'-3' nucleotide sequence	Indels
HEK-IP3R1	*IP3R3*	ATGAGTGAAATGTCCAGCTTTCTTCACATCGGGGACATCGTCTCCCTGTACGCCGAGGGCTCCGTCAATGGCTTCATCAGCACTTTGGG	WT
		ATGAGTGAAATGTCCAGCTTTCTTCA--TCAGCACTTTGGG	-50 bp
		ATGAGTGAAATGTCCAGCTTTCTTCA--TCGGGGACATCGTCTCCCTGTACGCCGAGGGCTCCGTCAATGGCTTCATCAGCACTTTGGG	-2 bp
	IP3R2	ATGACTGAGAAAATGTCCAGCTTCCTCTACATAGGGGACATCGTGTCCCTGTACGCGGAGGGCTCGGTCAACGGCTTCATCAGCACCTTGGG	WT
		ATGACTGAGAAAATGTCCAGCTTCCTCTACATAGGGGACATCGTGTCCCTGT--------GGCTCGGTCAACGGCTTCATCAGCACCTTGGG	-8 bp
		ATGACTGAGAAAATGTCCAGCTTCCTCTACATAGGGGACATCGTGTCCCTGTTACGCGGAGGGCTCGGTCAACGGCTTCATCAGCACCTTGGG	+1 bp
	IP3R1	ATGTCTGACAAAATGTCTAGCTTCCTACATATTGGAGACATTTGTTCTCTGTACGCGGAGGGATCGACAAATGGATTTATTAGCACCTTGG	WT
		ATGTCTGACAAAATGTCTAGCTTCCTACATATTGGAGACATTTGTTCTCTGTACGCGGAGGGATCGACAAATGGATTTATTAGCACCTTGG	0
HEK-IP3R2	*IP3R1*	ATGTCTGACAAAATGTCTAGCTTCCTACATATTGGAGACATTTGTTCTCTGTACGCGGAGGGATCGACAAATGGATTTATTAGCACCTTGG	WT
		ATGTCTGACAAAATGTCTAGCTTCCTACATATTGGAGACATTTGTTCTCTGTACGCGGAGGGATCGACAAATGGATTTATTAGTTATTAGCACCTTGG	+7 bp
		ATGTCTGACAAAATGTCTAGCTTCCTACATATTGGAGACATTTGTTCTCTGTACGCGGAGGGATCGACAAATGGATTTATTAGTTATTAGCACCTTGG	+7 bp
	IP3R3	ATGAGTGAAATGTCCAGCTTTCTTCACATCGGGGACATCGTCTCCCTGTACGCCGAGGGCTCCGTCAATGGCTTCATCAGCACTTTGGG	WT
		ATGAGTGAAATGTCCAGCTTTCTTCA--TCGGGGACATCGTCTCCCTGTACGCCGAGGGCTCCGTCAATGGCTTCATCAGCACTTTGGG	-2 bp
		ATGAGTGAAATGTCCAGCTTTCTTCA--TCGGGGACATCGTCTCCCTGTACGCCGAGGGCTCCGTCAATGGCTTCATCAGCACTTTGGG	-2 bp
	IP3R2	ATGACTGAGAAAATGTCCAGCTTCCTCTACATAGGGGACATCGTGTCCCTGTACGCGGAGGGCTCGGTCAACGGCTTCATCAGCACCTTGGG	WT
		ATGACTGAGAAAATGTCCAGCTTCCTCTACATAGGGGACATCGTGTCCCTGTACGCGGAGGGCTCGGTCAACGGCTTCATCAGCACCTTGGG	0
HEK-IP3R3	*IP3R2*	ATGACTGAGAAAATGTCCAGCTTCCTCTACATAGGGGACATCGTGTCCCTGTACGCGGAGGGCTCGGTCAACGGCTTCATCAGCACCTTGGG	WT
		ATGACTGAGAAAATGTCCAGCTTCCTCTACATAGGGGACATCGTGTCCCTGT----------CTCGGTCAACGGCTTCATCAGCACCTTGGG	-10 bp
		ATGACTGAGAAAATGTCCAGCTTCCTCTACATAGGGGACATCGTGTCCCTGTTACGCGGAGGGCTCGGTCAACGGCTTCATCAGCACCTTGGG	+1 bp
	IP3R1	ATGTCTGACAAAATGTCTAGCTTCCTACATATTGGAGACATTTGTTCTCTGTACGCGGAGGGATCGACAAATGGATTTATTAGCACCTTGG	WT
		ATGTCTGACAAAATGTCTAGCTTCCTACATATTGG------------------------------------GATTTATTAGCACCTTGG	-38 bp
		ATGTCTGACAAAATGTCTAGCTTCCTACATATTGGAGACATTTGTTCTCTGTACGCGGAG-----------------------------	-238 bp
	IP3R3	ATGAGTGAAATGTCCAGCTTTCTTCACATCGGGGACATCGTCTCCCTGTACGCCGAGGGCTCCGTCAATGGCTTCATCAGCACTTTGGG	WT
		ATGAGTGAAATGTCCAGCTTTCTTCACATCGGGGACATCGTCTCCCTGTACGCCGAGGGCTCCGTCAATGGCTTCATCAGCACTTTGGG	0

*The insertions are shown in red.

II. Expression analysis

In the experiments described below, expression of certain genes in WT-HEK cells and their derivatives was analyzed by both RT-PCR and RT-qPCR approaches. Total RNA was isolated from a particular cell colony (~10^6 cells), using Gen Elute Mammalian Total RNA Miniprep Kit (Sigma-Aldrich, St. Louis, MO, USA) in line with manufacturer protocol. Traces of genomic DNA in RNA preparations were degraded with DNAse I (New England Biolabs, Ipswich, MA, USA). Reverse transcription was performed using SuperScript IV VILO Master Mix (Thermo Fisher Scientific, Waltham, MA, USA). PCR amplification of targeted transcripts was performed using Phusion Hot Start II High-Fidelity DNA Polymerase (Thermo Fisher Scientific, Waltham, MA, USA) and gene-specific primers (Table 1S). Consistently with earlier reports (e.g. Alzayady et al., 2016), it was found that WT- HEK cells express all three isoforms (Fig,1SA).

Quantitative expression analysis was performed using RT-qPCR reaction mix Maxima SYBR Green qPCR Master Mix (Thermo Fisher Scientific, Waltham, MA, USA), and a real-time PCR instrument DTlight (DNA Technology, Protvino, Russia). Amplifications were performed starting with a 10 min template denaturation step at 95° C, followed by 40 cycles of denaturation at 95° C for 15 s, primer annealing at the gene specific primer temperature for 30 s and extension at 30° C for 30 s with a simultaneous fluorescence readout. Each sample was amplified in triplicates at least, and the averaged readout was used for further analysis. Levels of gene expression were evaluated by the $2^{-\Delta Ct}$ method with actin gene being a reference.

II.1. Expression of IP_3- and muscarinic receptors and annexin A1 in WT-HEK cells.

We analyzed expression of IP_3Rs in WT-HEK cells by using both RT-PCR and RT-qPCR. In line with previous studies (e.g. Alzayady et al., 2016), it was found that WT- HEK cells express all three isoforms (Fig,6SA). The quantitative analysis revealed that the relative level of *IP3R2* transcripts exceeded those obtained for *IP3R1* и *IP3R3* by nearly 10- and 2 times, respectively (Fig.6SB)

Mammalian genomes contains five genes encoding muscarinic receptors of distinct subtypes denoted M1 to M5 (Kruse et al., 2014). Using ordinary RT-PCR and gene specific primers, we identified M1-, M3-, M4-, and M5- transcripts in WT-HEK cells, while M2-transcripts were undetectable (Fig.7S, A). As showed by the qPCR analysis, the level of M3-trtanscripts exceeded the others by almost two orders of magnitude, indicating M3 to be the predominant isoform (Fig.7SB).

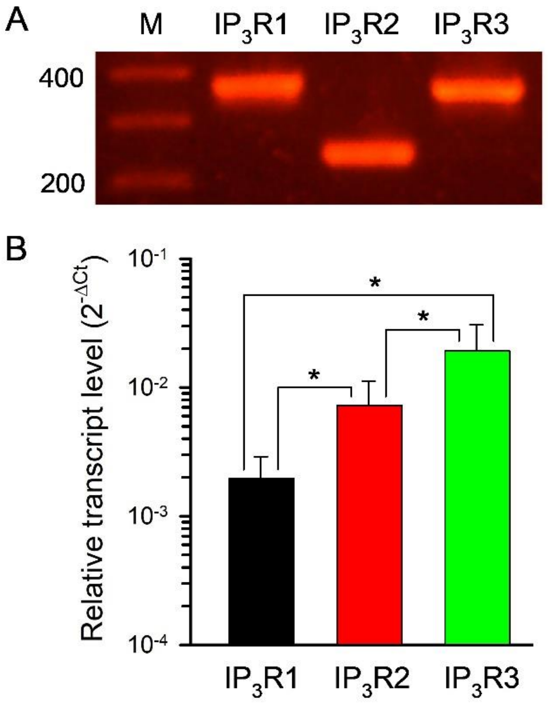

Figure 6S. Expression of IP_3Rs in WT-HEK cells. (A) Representative RT-PCR analysis (n=3) of expression of human IP_3Rs in WT-HEK cells. The detected transcripts of the IP_3R1-, IP_3R2- и IP_3R3-isoforms are seen as amplicons of the expected sizes of 379, 347, and 218 bp, respectively. The molecular weight markers (M) as in Fig.2S. The amplicons were visualized by EtBr in 1.2% agarose gel. Here and below, relative levels of *IP3R1*, *IP3R2* и *IP3R3* transcripts in HEK-293 cells (B) were calculated as $2^{-\Delta Ct}$ with actin transcripts taken as referent. The data are presented as a mean±S.D. (n=6). The asterisks mark statistically significant difference (p < 0.001, Anova test)

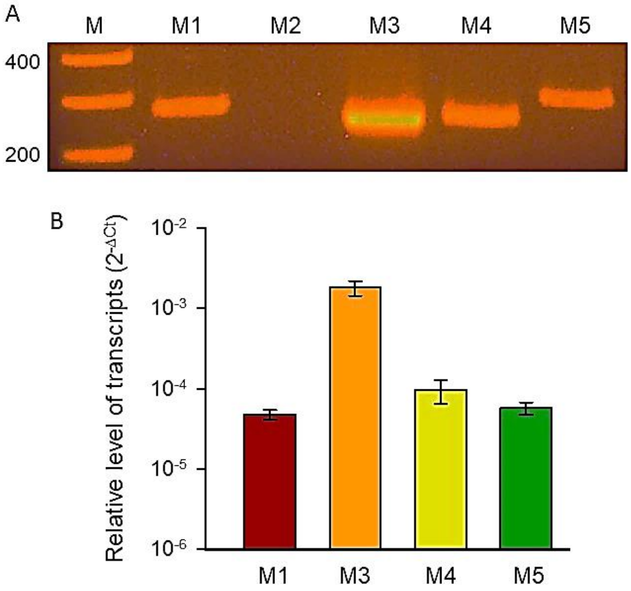

Figure 7S. Expression of muscarinic receptors in WT-HEK cells. (A) Representative RT-PCR analysis (n=3) of expression of human muscarinic receptors in WT-HEK cells. The transcripts of expected sizes were detected for M1- (278 bp), M3- (256 bp), M4- (261 bp), and M5-receptors (310 bp), while M2 transcripts (262 bp) were not detectable. The molecular weight markers (M) were as in Fig.2S. The amplicons were visualized by EtBr in 1.2% agarose gel. (B) Relative levels of M1-, M3-, M4-, and M5- transcripts in WT-HEK cells. The data are presented as a mean±S.D. (n=3).

The expression of the annexin A1 gene was analyzed by RT-PCR with gene specific primers (Table 2S). The triplicated analysis revealed *ANXA1* transcripts in RNA preparations isolated from WT-HEK cell colonies (Fig.8S), indicating that annexin A1 is expressed in WT-HEK cells indeed.

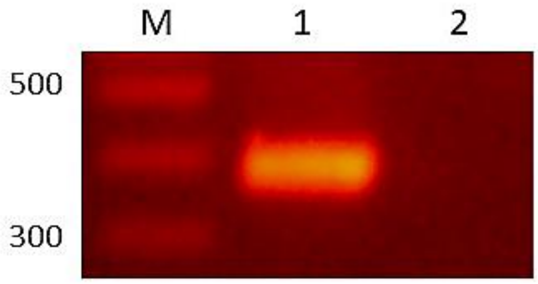

Figure 8S. Representative RT-PCR analysis (n=3) of expression of human annexin A1 in WT-HEK cells. PCR product of expected sizes (291 bp) was detected for RT-PCR with gene specific primers (lane 1). No amplification was detected in negative control PCR reaction without reverse transcription (lane 2). The molecular weight markers (M) were as in Fig.2S. The amplicon and markers were visualized by EtBr in 1.2% agarose gel.

II.3. Expression of signaling proteins in different cell lines.

Reportedly, genome editing with RNA–guided nucleases, such as Cas9, is associated with off-target DNA cleavage (Fu et al., 2013; Cho et al., 2014). We therefore analyzed the expression of muscarinic receptors and several downstream signaling proteins that could potentially be involved in ACh transduction, including G_q- and $G_{i1\text{-}3}$-proteins that couples a variety of GPCRs to G-protein-regulated PLC$\beta_{1\text{-}4}$ (Kadamur, Ross, 2013). The relative levels of their expression were evaluated by using qRT-PCR with the actin gene as a reference.

While the expression of M-receptors exhibited some variability across different lines, the average transcript levels of a specific M-isotype were statistically indistinguishable (ANOVA test), with few exceptions (Fig.9S). Specifically, a level of M1- transcripts in IP_3R3-HEK cells was nearly twofold lower compared to one in IP_3R1-HEK- and IP_3R2-HEK cells (Fig.8SA, panel M1); expression of the M5-receptor was twice higher in IP_3R2-HEK cells compared to WT-HEK cells (Fig.8SA, panel M5). Note that our findings suggested that just M3-receptor was central to ACh-induced Ca^{2+} mobilization in assayed cells (Fig.6C, D and Fig.7S, B). Given this fact and statistically indistinguishable levels of M3-transcripts in IP_3R1-, IP_3R2-, and IP_3R3-HEK cells (Fig.3, panel M3), it appeared that the mechanism responsible for distinct sensitivities of the assayed cell lines to ACh (Fig.2B) likely lay downstream of the receptor level.

The M3-receptor couples primarily to the G_q-protein (Saternos et al., 2018), and therefore, its level in the particular cell line could be a key factor that determined ACh-dependent IP_3 production and the related dose-response curve (Fig.2B). WT-HEK-, IP_3R2-HEK-, and IP_3R3-HEK cells showed statistically indistinguishable levels of G_q expression (Fig.9S,B, G_q panel), although for the IP_3R1-HEK/IP_3R3-HEK pair, there was a statistically significant difference of about 50% between the levels of G_q transcripts. Nevertheless, the observed pattern of G_q transcripts (Fig.9S,B, G_q panel) did not support the idea of a strong

correlation between G_q expression in the particular cell line and its sensitivity to ACh (Fig.2B). In addition, we found that in all four cell lines, levels of $G_{i1\text{-}3}$ transcripts were close and statistically indistinguishable (Fig.9S,B, G_i panels).

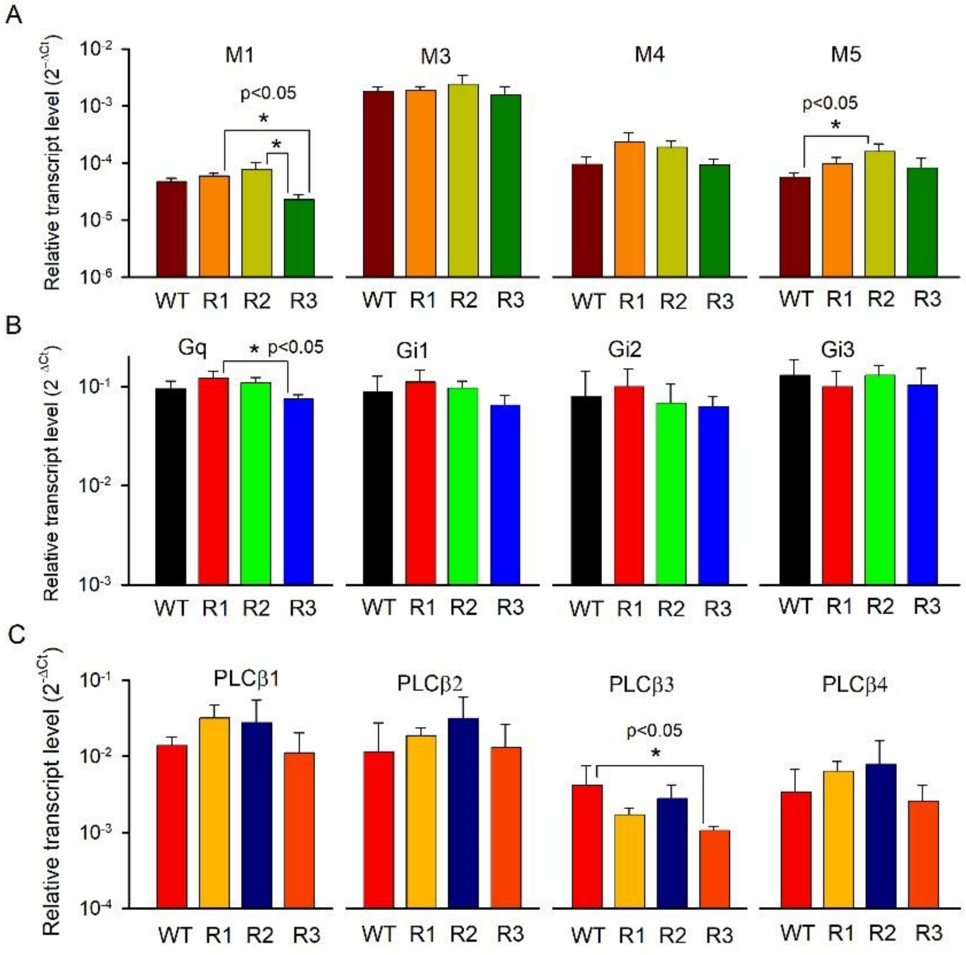

Figure 9S. Expression of signaling proteins in cells of different lines.

A-C. Relative levels of transcripts of muscarinic receptors (A), Gq-and Gi-proteins (B), and G-protein regulated PLCs (C) in WT-, IP_3R1-, IP_3R2-, and IP_3R3-HEK cells. The asterisks mark statistically significant difference (ANOVA test, $p < 0.05$).

Although expression of PLCβ1-4 was more scattered among cell populations, statistically significant deviations exhibited solely PLCβ3 (Fig.9S, C). Given however that compared to PLCβ1 and PLCβ2, the level of PLCβ3 transcripts was lower by the factor 3-10 (Fig.9S, C), this PLC isoform presumably provided a minor contribution to ACh signaling (Fig.2).

We also questioned whether gene editing and cell selection could lead to a change in expression of IP_3Rs in engineered cell lines, compared to WT-HEK cells. The only reliable increase in IP3R expression, that is IP_3R3, was found in IP_3R3-HEK cells, while in the WT-

HEK/IP_3R1-HEK and WT-HEK/IP_3R2-HEK pairs, expression of IP_3R1 and IP_3R2, respectively, was at statistically indistinguishable levels (Fig.10S).

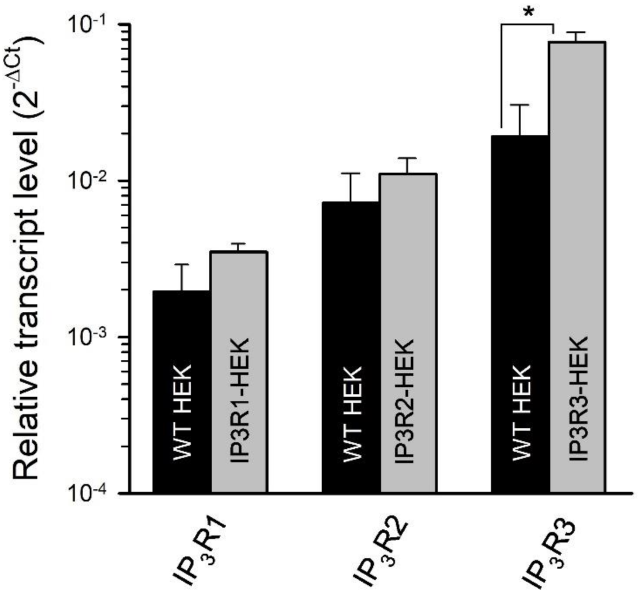

Figure 10S. Expression of IP_3Rs in cells of different lines.

Relative levels of transcripts of IP3R isoforms in WT-, IP_3R1-, IP_3R2-, and IP_3R3-HEK cells. The statistically significant difference is marked by the asterisk ($p < 0.01$, Student t-test).

Table 2S. Primer sequences*

Targeted gene	Protein	5'-sequence-3'		T_m** (ºC)	Product size (bp)
ITPR1	IP3R1	F	GTCGGAATTAAAGGATCAGATGAC	53,4	377
		R	AAGTGCAAATCAGGTGCTTTC	54,1	
ITPR2	IP3R2	F	AAAGACCCAACAGAATACACTG	52,8	237
		R	ATTCTTCCTTTGTTCTGTCATCTG	53,2	
ITPR3	IP3R3	F	GACATGCTTCATCTGTGGTC	53,5	358
		R	TTTCCGCTGCTCCGTCATC	57,9	
CHRM1	CHRM1	F	CCT ACA GAC CCC TCT TCA GC	56,9	278
		R	TGT CTT GAG CTC CGT GTT GAC C	59,3	
CHRM2	CHRM2	F	CGAAACCAGCGACAGGTTGT	58,1	262
		R	GTTCCCGATAATGGTCACCAAAC	56,1	
CHRM3	CHRM3	F	GTC CTA TGC CGG GAT CAT CAT G	57,5	256
		R	GGA GAG GAG AAA TTG CCA GCT G	58	
CHRM4	CHRM4	F	AGCAGTGACGATGGCCTG	57,8	261
		R	TTGGTGCCAATTCTGATGCATG	56,5	
CHRM5	CHRM5	F	CTTACCACAATGCAACCACC	54,3	310
		R	GCAAGCCAAAGGTCACAAGC	57,6	

GNAQ	G_q	F	CAAGTTCTCGTGGAGTCAGAC	55,1	197
		R	GCATCTCTCTGGGGTCCATC	57,3	
GNAI1	G_{i1}	F	CGACCTGGTTCTAGCTGAAGATG	57,1	213
		R	GCAGCTGCCTCTTCATATGTG	56,2	
GNAI2	G_{i2}	F	CTGAGGACGAGGAGATGAACC	56,7	215
		R	CTTACTCTGGATGTAGCTGGC	54,9	
GNAI3	G_{i3}	F	GCAGCTGCCTATATTCAATGCC	56,6	223
		R	GTCTGGTCTCAACACTCCACAC	57,5	
PLCB1	PLCβ1	F	GGATGTCGCAGAAGAGTGTC	55,4	226
		R	ACTTTGCGCTTCTTCTAGCC	55,5	
PLCB2	PLCβ2	F	AAGATGGCCCAGGAGAGGTTG	59,2	235
		R	TCACCGACTCCTTCACCTCTG	58,3	
PLCB3	PLCβ3	F	GGCAAAGCGGTATCAGGAGTTC	58,1	204
		R	TGCAGCTCCTTCTTCTCCCTC	58,8	
PLCB4	PLCβ4	F	AAAGCTACTCATCAATGCCCAC	55,5	184
		R	GCTGTTTAACTCCCTGACTCGC	58	
ACTB	β-actin	F	CGCGAGAAGATGACCCAGATC	57,5	226
		R	TCATGAGGTAGTCAGTCAGGTCC	57,6	
ANXA1	annexin A1	F	ACAAAGTTCTGGACCTGGAGTTG	57,1	291
		R	TTCCTCCACAAAGAGCCACCAG	59,3	

*The primers were designed to be either intron-spanning or located in different exons in order to recognize all known splice variants of particular transcripts.

** Melting temperature T_m was calculated using OligoAnalyzer™ Tool (IDT).

II.4. Western blot assay of IP3R expression

Several experiments were designated to verify the expression data of IP_3R transcripts in engineered cells. In each case, cells (~10^7) were pelleted and directly transferred to 300 μL of 1x Laemmli buffer. Samples were incubated for 10 min at 95°C, and each probe (20 μL) was applied on 4-15% BIS-TRIS gradient gel. Protein transfer was performed on 0.45 μm nitrocellulose membranes using the PowerBlotter semi-dry transfer system (BioRad Laboratories, USA). Membranes were probed with primary antibodies to IP_3R1 (rabbit polyclonal antibodies against rat IP3R1 (2732–2750 aa, Alomone Labs, Israel, Cat# ACC-019), type 2 (rabbit polyclonal antibodies against rat IP_3R2 (2683-2696 aa, Alomone Labs, Israel, Cat# ACC-116) or human IP_3R3 (mouse monoclonal antibodies against 22-230 aa, BD Transduction Laboratories, USA, Cat# 610312). Next, probes were incubated with horseradish

peroxidase conjugated secondary antibodies Anti-Rabbit IgG Peroxidase Conjugate (Sigma-Aldrich, USA Cat# A-9169) and Anti-Mouse IgG, IgA, IgM Peroxidase Conjugate (IMTEC, Russia, Cat# P-GAM Iss). Anti-actin was from United States Biological (Cat# A0760-40). Blots were imaged using iBright™ CL750 Imaging System (Thermo Fisher Scientific, USA) and enhanced chemiluminescent substrate SuperSignal™ West Pico PLUS Chemiluminescent Substrate (Thermo Fisher Scientific, Waltham, USA, Cat#34580).

It should be noted that only anti-IP3R1 and anti-IP3R3 provided conclusive results (Fig.11S), while anti-IP3R2 antibodies resulted in a heavy unspecific binding at many different sizes (not shown). Although the Western blot analysis was not complete for the mentioned technical reasons, the results we obtained for IP_3R1 and IP_3R3 match our mRNA data (Fig.10S).

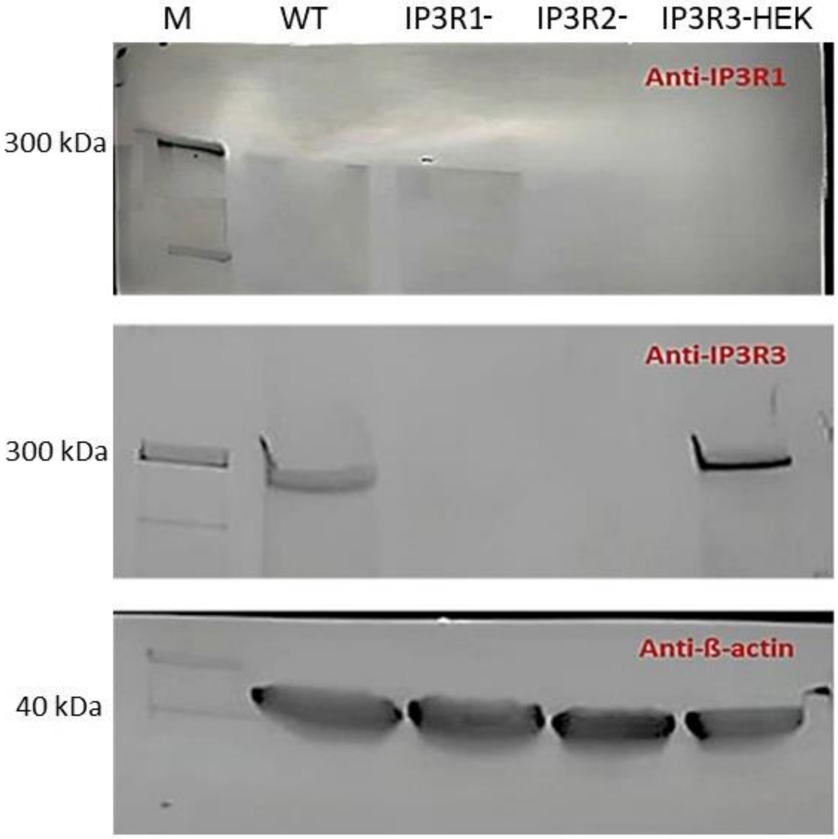

Figure 11S. Western blots from WT-, IP3R1-, IP3R2-, and IP3R3-HEK cells.

III. Ryanodine receptors contribute negligibly to Ca^{2+} leakage from ER.

The classical thapsigargin test was employed to evaluate a contribution of ryanodine receptors (RyRs) to Ca^{2+} leakage from ER in HEK-293 cells. The thapsigargin-induced Ca^{2+} signals were assayed in control and in the presence of micromolar ryanodine, a RyR inhibitor. Assayed cells were initially stimulated with 1 μM ACh, and Ca^{2+} homeostasis in a given cell was considered

as sufficiently robust, if its Ca^{2+} response to the agonist was fast and exceeded 2, in terms of $\Delta F/F_0$ (Fig.11SA). In control, cells were treated with 1 μM thapsigargin with 260 nM Ca^{2+} in the bath to make a contribution of Ca^{2+} entry to intracellular Ca^{2+} signals negligible. Under these conditions, thapsigargin-elicited Ca^{2+} transients were produced by Ca^{2+} leakage from ER, which emptied Ca^{2+} store and stimulated activity of SOCs, albeit SOCE was not evident at low bath Ca^{2+}. Thapsigargin was applied for 600 s, the interval that was rather sufficient for intracellular Ca^{2+} to return to the initial level. The restoration of bath Ca^{2+} to 2 mM initiated essential SOCE that resulted in marked Ca^{2+} responses (Fig.11SA). In a number of experiments, cells were first preincubated with 30 μM ryanodine and then treated with thapsigargin, and the inhibition of RyR activity was not associated with evident effects on the thapsigargin-induced Ca^{2+} release (Fig.11SB). To quantify one, Ca^{2+} traces from individual cells were differentiated, and maximal rates of Ca^{2+} release were determined as an appropriate local maximum in $d(F/F_0)/dt$ curves, as shown in Fig.3B-E. It turned out that the rates of thapsigargin-induced Ca^{2+} release in control and in the presence of ryanodine were statistically indistinguishable (Student t-test, $p<0.01$) (Fig.12SC), suggesting a negligible contribution of RyRs to Ca^{2+} leakage from ER.

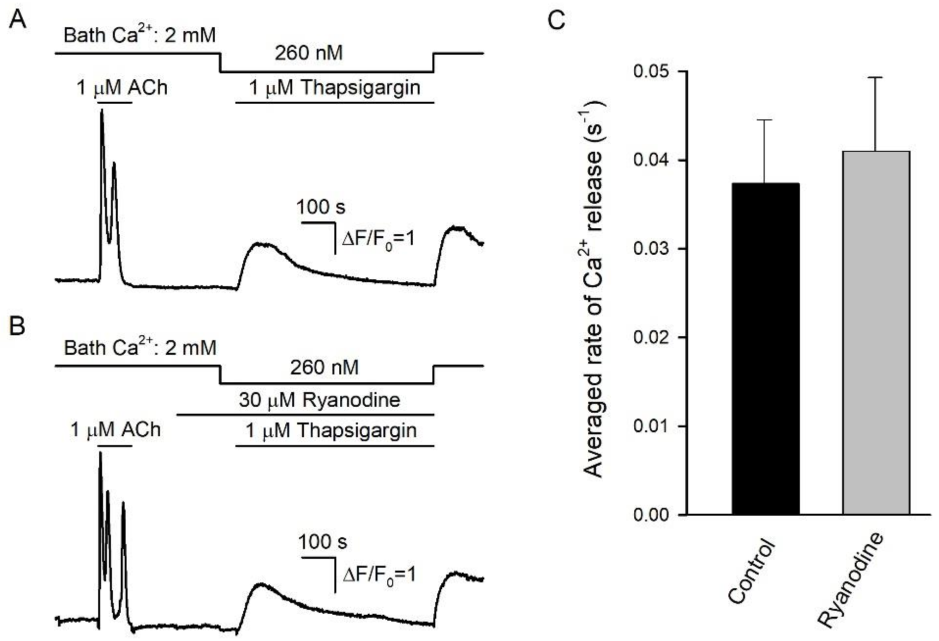

Figure 12S. Ca^{2+} signals associated with SERCA inhibition and Ca^{2+} store depletion by thapsigargin. (A, B) Representative Ca^{2+} transients elicited in WT-HEK cells in control (n=149) (A) and in the presence of 30 μM ryanodine (n=110) (B). (C) There was no statistically significant difference between averaged rates of thapsigargin-induced Ca^{2+} release in control and in the presence of 30 μM ryanodine. The data are presented as a mean±S.D (n=149/110 in control/ryanodine)

IV. Simplified model of Ca^{2+} homeostasis

We suggest here that in unstimulated cells, four main Ca^{2+} fluxes determine a level of cytosolic Ca^{2+}: entry of external Ca^{2+} through diverse Ca^{2+} permeable channels ($\boldsymbol{J}_{en}$); Ca^{2+} extrusion flux ($\boldsymbol{J}_{Ex}$) mediated by plasmalemmal Ca^{2+}-ATPase (PMCA) and/or Na^{+}/Ca^{2+} exchanger; Ca^{2+} leakage ($\boldsymbol{J}_{L}$) from endoplasmic reticulum (ER) through Ca^{2+} leak channels and spontaneously active IP_3Rs and RyRs; Ca^{2+} influx ($\boldsymbol{J}_{SERCA}$) to ER mediated by reticular Ca^{2+}-ATPase (SERCA) (Fig.10S). In the cytosol and ER, Ca^{2+} is subjected to binding to Ca^{2+} buffer, which is supposed to be fast and non-saturated, i.e. linear, so that concentrations of bound and free Ca^{2+} were proportional: $[Ca^{2+}]_b = \beta[Ca^{2+}]_f$. It therefore could be written for total Ca^{2+}: $[Ca^{2+}]_t=[Ca^{2+}]_b+[Ca^{2+}]_f=(1+\beta)[Ca^{2+}]_f \approx \beta[Ca^{2+}]_f$, given that β usually exceeds 100 (Neher, 1995).

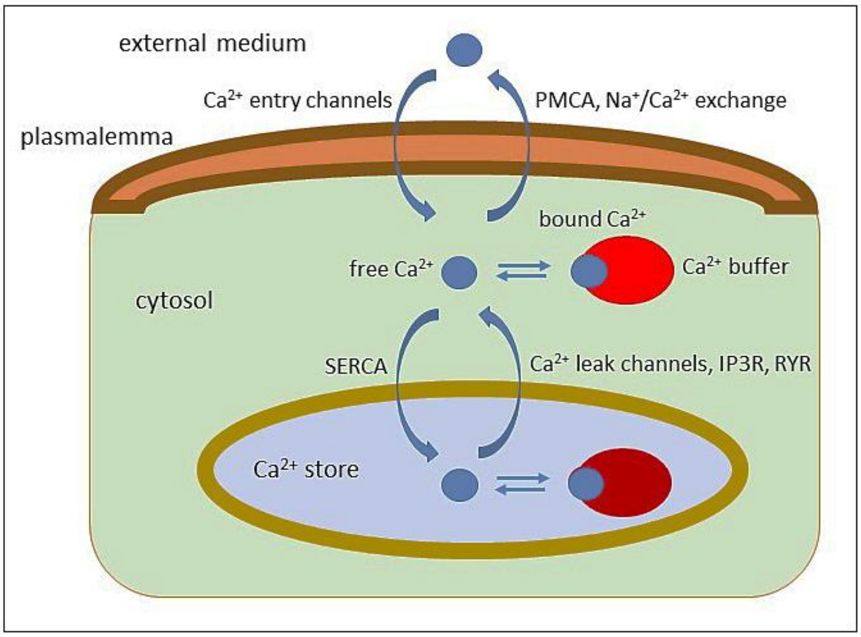

Figure.13S. Kinetics model of Ca^{2+} homeostasis in a unstimulated cell.

By introducing the variables C and C_S, which means the concentrations of free Ca^{2+} in the cytosol and reticular Ca^{2+} store, respectively, the following equations for Ca^{2+} balance can be written:

$$\alpha_C \frac{dC}{dt} = J_{En} + J_L - J_{Ex} - J_{SERCA} \qquad (1S)$$

$$\alpha_R \frac{dC_R}{dt} = J_{SERCA} - J_L \qquad (2S)$$

where $\alpha_C=\beta_C V_C$ and $\alpha_R=\beta_R V_R$ with β_C and β_R being Ca^{2+} binding coefficients of cytosolic and reticular Ca^{2+} buffers, V_C and V_R being volumes of the cytosol and reticular Ca^{2+} store. When

SERCA is inhibited by thapsigargin and J_{En} is nullified by removal of bath Ca^{2+}, the above Eqs reduced to:

$$\alpha_C \frac{dC}{dt} = J_L - J_{Ex} \qquad (3S)$$

$$\alpha_R \frac{dC_R}{dt} = -J_L \qquad (4S)$$

Because the linear approximation markedly simplified the analysis, we used the following expressions for Ca^{2+} fluxes: $J_L = P(C_R\text{-}C)$ and $J_{Ex}=QC$ where P is the total permeability of the ER membrane to Ca^{2+}, Q is a rate constant. At this approximation, Eqs 3S and 4S give:

$$\alpha_C \frac{dC}{dt} = P(C_R - C) - QC \qquad (5S)$$

$$\alpha_R \frac{dC_R}{dt} = -P(C_R - C) \qquad (6S)$$

By integrating Eq.5S over time from 0 to T, the interval of the cell exposure to thapsigargin, in the end of which cytosolic Ca^{2+} returned apparently to the initial level (Fig.5A), it can be derived:

$$\int_0^T \frac{dC}{dt} dt = C(T) - C(0) = 0 = \frac{P}{\alpha_C}\int_0^T C_R\, dt - \frac{P+Q}{\alpha_C}\int_0^T Cdt \quad (7S)$$

$$\int_0^T \frac{dC_R}{dt} dt = C_R(T) - C_R(0) = -\frac{P}{\alpha_R}\left(\int_0^T C_R dt - \int_0^T Cdt\right) \qquad (8S)$$

Suggesting that thapsigargin emptied ER significantly during the period T, $C_R(T) \ll C_R(0)$, and taking into account Eq (7S), it can be written:

$$\int_0^T Cdt = \frac{\alpha_R}{Q} C_R(0) \qquad (9S)$$

Since in a resting cell, $C_R \gg C$ at t=0, Eq.9S gives:

$$\frac{dC}{dt}(0) \approx \frac{P}{\alpha_C} C_R(0) = \frac{PQ}{\alpha_C \alpha_R}\int_0^T Cdt \qquad (10S)$$

that gives:

$$P \approx \frac{\alpha_C \alpha_R}{Q} \frac{\frac{dC}{dt}(0)}{\int_0^T Cdt} \qquad (11S)$$

References

Alzayady K.J.,Wang L., Chandrasekhar R.,Wagner L.E., II, Van Petegem F., Yule D.I. (2016). Defining the stoichiometry of inositol 1,4,5-trisphosphate binding required to initiate Ca^{2+} release. Sci. Signal. 9, ra35.

Cho S.W., Kim S., Kim Y., Kweon J., Kim H.S., Bae S., Kim J.S.(2014) Analysis of off-target effects of CRISPR/Cas-derived RNA-guided endonucleases and nickases. Genome Res. 24, 132-141.

Fu Y., Foden J.A., Khayter C., Maeder M.L., Reyon D., Joung J.K., Sander J.D. (2013) High-frequency off-target mutagenesis induced by CRISPR-Cas nucleases in human cells. Nat. Biotechnol. 31, 822-826.

Kadamur G., Ross E.M. (2013) Mammalian Phospholipase C. Annu. Rev. Physiol. 75, 127–154.

Kruse A., Kobilka B., Gautam D., Sexton P.M., Christopoulos A., Wess J. (2014) Muscarinic acetylcholine receptors: novel opportunities for drug development. Nat. Rev. Drug Discov. 13, 549–560.

Neher E. (1995) The use of Fura-2 for estimating Ca buffers and Ca fluxes. Neuropharm. 11, 1423-1442.

Saternos H.C., Almarghalani D.A., Gibson H.M., Meqdad M.A., Antypas R.B., Lingireddy A., AbouAlaiwi W.A. (2018) Distribution and function of the muscarinic receptor subtypes in the cardiovascular system. Physiol. Genomics. 50, 1-9.
